# Supplementary material for: Clinical effectiveness and cost effectiveness of individual mental health workers colocated within primary care practices: a systematic literature review
Source: BMJ Open. 2020 Dec 2;10(12):e042052. doi: 10.1136/bmjopen-2020-042052 (PMC7713190; doi:10.1136/bmjopen-2020-042052)
Supplement: Supplementary data [file bmjopen-2020-042052supp002.pdf]

Supplement 2: MEDLINE search strategy

1. exp Mental Disorders/ or mental disease*.mp. or exp Depression/ or mental disorder*.mp. or mental illness*.mp. or mental health illness*.mp.
2. mental disorder*.mp. [mp=title, abstract, original title, name of substance word, subject heading word, keyword heading word, protocol supplementary concept word, rare disease supplementary concept word, unique identifier]
3. Exp Mental Health/ or mental health problem*.mp. or exp Stress, Psychological/
4. stress.mp.
5. exp Depression/
6. depression.mp. [mp=title, abstract, original title, name of substance word, subject heading word, keyword heading word, protocol supplementary concept word, rare disease supplementary concept word, unique identifier]
7. exp Anxiety/ or anxiety.mp. or exp Anxiety Disorders/ or anxiety disorder*.mp.
8. (nervous breakdown$ or depression or depressive$ or affective disorder$ or mood disorder$ or anxiety disorder$ or phobia or phobic or panic disorder$ or PTSD or stress disorder$ or OCD or compulsive disorder$).mp. [mp=title, abstract, original title, name of substance word, subject heading word, keyword heading word, protocol supplementary concept word, rare disease supplementary concept word, unique identifier]
9. (anxi* or depress* or melancholi* or neuros* or neurotic or psychoneuro* or stress* or distress* or emotion*).mp.
10. ((mental$ or psychiatric or psychologic$) adj3 (illness$ or condition$ or disabil$ or disorder$ or disease$ or impair$ or problem)).mp. [mp=title, abstract, original title, name of substance word, subject heading word, keyword heading word, protocol supplementary concept word, rare disease supplementary concept word, unique identifier]
11. (bipolar or mania or dissociative disorder$ or neurosis or personality disorder$ or psychosis or paranoi$ or schizophren$ or schizoaffective disorder$ or psychotic).mp. [mp=title, abstract, original title, name of substance word, subject heading word, keyword heading word, protocol supplementary concept word, rare disease supplementary concept word, unique identifier]
12. 1 or 2 or 3 or 4 or 5 or 6 or 7 or 8 or 9 or 10 or 11
13. (Primary adj2 (care or health or healthcare)).mp. [mp=title, abstract, original title, name of substance word, subject heading word, keyword heading word, protocol supplementary concept word, rare disease supplementary concept word, unique identifier]
14. primary health care/ or *continuity of patient care*/ or patient-centred care or progressive patient care/
15. (Primary pratic* or Family pratic* or General practice*).mp. [mp=title, abstract, original title, name of substance word, subject heading word, keyword heading word, protocol supplementary concept word, rare disease supplementary concept word, unique identifier]
16. Community Health Services/
17. community Health Service*.mp. [mp=title, abstract, original title, name of substance word, subject heading word, keyword heading word, protocol supplementary concept word, rare disease supplementary concept word, unique identifier]
18. exp Community Mental Health Services/ or community care.mp. or Community Mental Health Service.mp. [mp=title, abstract, original title, name of substance word, subject heading word, keyword heading word, protocol supplementary concept word, rare disease supplementary concept word, unique identifier]
19. exp Family Practice/ or exp General Practice/
20. 13 or 14 or 15 or 16 or 17 or 18 or 19
21. (mental health adj2 (nurs* or therap* or counsellor* or worker* or practitioner*)).mp. [mp=title, abstract, original title, name of substance word, subject heading word, keyword heading word, protocol supplementary concept word, rare disease supplementary concept word, unique identifier]
22. psychotherapist.mp.
23. therapist*.mp.
24. counsellor*.mp.
25. 21 or 22 or 23 or 24

26. co-locat*.mp. [mp=title, abstract, original title, name of substance word, subject heading word, keyword heading word, protocol supplementary concept word, rare disease supplementary concept word, unique identifier]
27. colocat*.mp. [mp=title, abstract, original title, name of substance word, subject heading word, keyword heading word, protocol supplementary concept word, rare disease supplementary concept word, unique identifier]
28. integrat*.mp. [mp=title, abstract, original title, name of substance word, subject heading word, keyword heading word, protocol supplementary concept word, rare disease supplementary concept word, unique identifier]
29. ((integrat or within or collaborat* or colocat* or co-locat* or inside) adj primary health care).mp. [mp=title, abstract, original title, name of substance word, subject heading word, keyword heading word, protocol supplementary concept word, rare disease supplementary concept word, unique identifier]
30. ((integrat or within or collaborat* or colocat* or co-locat* or inside) adj primary care).mp. [mp=title, abstract, original title, name of substance word, subject heading word, keyword heading word, protocol supplementary concept word, rare disease supplementary concept word, unique identifier]
31. ((integrat or within or collaborat* or colocat* or co-locat* or inside) adj primary healthcare).mp. [mp=title, abstract, original title, name of substance word, subject heading word, keyword heading word, protocol supplementary concept word, rare disease supplementary concept word, unique identifier]
32. exp "Delivery of Health care, Integrated*/ or integrated care.mp. or integrated healthcare.mp. or integrated health care.mp.
33. collaborat*.mp. [mp=title, abstract, original title, name of substance word, subject heading word, keyword heading word, protocol supplementary concept word, rare disease supplementary concept word, unique identifier]
34. 26 or 27 or 28 or 29 or 30 or 31 or 32 or 33
35. 12 and 20 and 25 and 34
