# Supplementary material for: Clinical effectiveness and cost effectiveness of individual mental health workers colocated within primary care practices: a systematic literature review
Source: BMJ Open. 2020 Dec 2;10(12):e042052. doi: 10.1136/bmjopen-2020-042052 (PMC7713190; doi:10.1136/bmjopen-2020-042052)
Supplement: Supplementary data [file bmjopen-2020-042052supp003.pdf]

Supplement 3: Bias levels in non-randomised studies. Obtained via utilization of the Robins-I tool(15).

	Confounding bias	Bias in selection of participants	Bias in classification of interventions	Bias due to deviations from intended interventions	Bias due to missing data	Bias in measurement of outcomes	Bias in selection of the reported result
Kates et al., (27)	?	?	+	?	+	+	+
Cigrang et al., (26)	−	+	+	+	?	?	+
Abidi et al., (29)	?	+	+	+	?	+	+
Evans et al., (30)	?	?	?	+	?	+	+
Milne and Souter (40)	+	?	+	+	−	+	+
Bridges et al., (38)	+	+	+	+	−	−	+
Magnée et al., (34)	+	+	+	?	?	?	+
Pryde and Jachuck (32)	?	?	+	+	?	?	+
Spurgeon et al., (31)	+	−	?	+	−	+	+
Magnée et al., (33)	?	+	+	+	−	−	+
