# Supplementary material for: Clinical effectiveness and cost effectiveness of individual mental health workers colocated within primary care practices: a systematic literature review
Source: BMJ Open. 2020 Dec 2;10(12):e042052. doi: 10.1136/bmjopen-2020-042052 (PMC7713190; doi:10.1136/bmjopen-2020-042052)
Supplement: Supplementary data [file bmjopen-2020-042052supp004.pdf]

Supplement 4: Bias levels in randomised controlled trials. Obtained via utilization of the Cochrane bias tool(16).

	Random sequence generation	Allocation concealment	Blinding of participants and personnel	Blinding of outcome assessment	Incomplete outcome data	Selective reporting	Anything else, ideally prespecified
Boot et al., (28)	+	−	−	−	?	?	?
McMahon et al., (35)	?	?	−	+	−	+	?
Lester et al., (36)	+	+	−	?	+	+	?
Marks (37)	?	?	−	?	−	+	?
Friedli, King and Lloyd (39)	?	?	−	−	?	?	?
