# Supplementary material for: Clinical effectiveness and cost effectiveness of individual mental health workers colocated within primary care practices: a systematic literature review
Source: BMJ Open. 2020 Dec 2;10(12):e042052. doi: 10.1136/bmjopen-2020-042052 (PMC7713190; doi:10.1136/bmjopen-2020-042052)
Supplement: Supplementary data [file bmjopen-2020-042052supp005.pdf]

Supplement 5: Bias assessment for randomised controlled trials, summary of results.

Cochrane Risk of Bias Tool for Randomized Controlled Trials.

Author	Selection bias		Performance bias	Detection bias	Attrition bias	Reporting bias	Other bias	Total
	Random sequence generation	Allocation concealment	Blinding of participants and personnel	Blinding of outcome assessment	Incomplete outcome data	Selective reporting	Anything else, ideally prespecified	
Boot et al., (28)	Low	High	High	High	Unclear	Unclear	Unclear	Poor quality
McMahon et al., (35)	Unclear	Unclear	High	Low	High	Low	Unclear	Fair quality
Lester et al., (36)	Low	Low	High	Unclear	Low	Low	Unclear	Good quality
Marks (37)	Unclear	Unclear	High	Unclear	High	Low	Unclear	Poor quality
Friedli, King and Lloyd (39)	Unclear	Unclear	High	High	Unclear	Unclear	Unclear	Poor quality
