# Supplementary material for: Clinical effectiveness and cost effectiveness of individual mental health workers colocated within primary care practices: a systematic literature review
Source: BMJ Open. 2020 Dec 2;10(12):e042052. doi: 10.1136/bmjopen-2020-042052 (PMC7713190; doi:10.1136/bmjopen-2020-042052)
Supplement: Supplementary data [file bmjopen-2020-042052supp006.pdf]

Supplement 6: Bias assessment in non-randomised studies of interventions, summary of results. Risk Of Bias In Non-randomized Studies of Interventions (ROBINS-I) tool.

Author	Pre-intervention		At intervention	Post intervention				Overall bias
	Confounding bias	Bias in selection of participants	Bias in classification of interventions	Bias due to deviations from intended interventions	Bias due to missing data	Bias in measurement of outcomes	Bias in selection of the reported result	
Kates et al., (27)	Unclear	Unclear	Low	Unclear	Low	Low	Low	Good Quality
Cigrang et al., (26)	High	Low	Low	Low	Unclear	Unclear	Low	Fair Quality
Abidi et al., (29)	Unclear	Low	Low	Low	Unclear	Low	Low	Good Quality
Evans et al., (30)	Unclear	Unclear	Uncertain	Low	Unclear	Low	Low	Unclear Quality
Milne and Souter (40)	Low	Unclear	Low	Low	High	Low	Low	Fair quality
Bridges et al., (38)	Low	Low	Low	Low	High	High	Low	Good quality
Magnée et al., (34)	Low	Low	Low	Unclear	Unclear	Unclear	Low	Good quality
Pryde and Jachuck (32)	Unclear	Unclear	Low	Low	Unclear	Unclear	Low	Unclear quality
Spurgeon et al., (31)	Low	High	Unclear	Low	High	Low	Low	Fair Quality
Magnée et al., (33)	Unclear	Low	Low	Low	High	High	Low	Fair Quality
