# Supplementary material for: Clinical effectiveness and cost effectiveness of individual mental health workers colocated within primary care practices: a systematic literature review
Source: BMJ Open. 2020 Dec 2;10(12):e042052. doi: 10.1136/bmjopen-2020-042052 (PMC7713190; doi:10.1136/bmjopen-2020-042052)
Supplement: Supplementary data [file bmjopen-2020-042052supp007.pdf]

Supplement 7: Summary of findings in individual studies

Author	Findings
Kates et al., (27)	Clinical: An average change in score of 17.6 (CESD) and 5.7 (GHQ) was recorded. Furthermore, after comparing the numbers of patients classified as a case (due to exceeding cut off score) before and after treatment, there was 73% (CESD) and 82% (GHQ) less cases after treatment. All changes statistically significant (P<0.005).<br>Service utilization: 65% reduction in referrals to psychiatry outpatient since program started. Nonsignificant reduction of 10% in inpatient admissions.<br>Satisfaction: Overall consumer satisfaction rating of 92% (CSQ). Counsellors and practitioners satisfied.
Cigrang˙ et al., (26)	Clinical: OQ-45 scores decreased significantly for patients with more than one appointment. There was also a significant reduction in OQ-45 scores between patients that had either had 1, 2 or 4 or more appointments. This was also maintained during follow up (P range 0.032-0.001)<br>Service utilization: Significant increase in medical appointments P<0.01.
Boot et al., (28)	Clinical: Post intervention GHQ scores were significantly reduced for both the counselling intervention group and a control GP advice group. However, GHQ scores were significantly lower in the intervention group than in the control group and the average decrease in scores was twice as high in intervention group. There was a significantly larger proportion of the control group scoring above the GHQ threshold level post study, 46% compared to 25%.<br>Psychotropic drug usage:<br>Significantly fewer prescriptions during the 6- week period in the counselled group compared to GP care. Similar finding for only antidepressants but not anxiolytic drugs.<br>Referrals and consultation rates: Significantly higher referrals in GP group. No significant differences in follow-up consultations rates.<br>Satisfaction: significantly more counselled patients reported coping better with their problem, feeling happier, satisfied with treatment and had enough time to talk.
Abidi et al., (29)	Diagnoses: significant decrease of chronic alcohol abuse and acute alcohol abuse diagnoses for all groups (control, only PN-MH, only PCP, both PCP and PNMH). both PCP and PNMH higher mean of chronic and acute alcohol abuse diagnoses than control practices. No significant differences were found between groups.
Evans et al., (30)	Referrals: mean referral rate of BME patients for GP practices without a link worker 0.35 per week per 10000 patients and was unchanged throughout study. Referral rates for practices with a link worker increased from 0.65 to 1.37 referrals per week per 10000 patients.
Spurgeon et al., (31)	Clinical outcomes: All patients showed reductions in subjective distress (CORE-OM), excluding the diabetes group, and was maintained post-intervention. The HADS anxiety and depression scales show there was significant reductions in anxiety in all patient groups, patients with hypertension and frequent attenders had reductions in depression also. The total patient sample achieved significant reductions in the majority of SF36 subscales, excluding diabetic patients<br>Uptake of health services: Counselling intervention significantly reduced uptake of health services
Pryde and Jachuck (32)	Clinical outcomes: After an average of three consultations, out of 84 patients, 44% were considered to be much improved, 31% improved and 25% unchanged in terms of symptomatic severity and prevalence.
Magnée et al., (34)	Service utilization: GPs in practices with a mental health nurse have similar consultation rates than GPs without one. GPS with a mental health nurse have more patients exhibiting psychological or social disorders (OR=1.05). Nurses do not seem to replace GP care, but probably deliver additional consultations to patient

McMahon et al., (35)	Clinical outcomes: Little difference between the two treatments BDI. Although, significant gradual symptomatic improvement was seen over time for this measure. Some improvement over time was seen for other measures (HDRS17, MADRS and SASS) but was not significant and could be as a result of intervention group patients being significantly more likely to have medication adjustment (P<0.01).<br>Satisfaction: Patient satisfaction high for both treatments.
Lester et al., (36)	Clinical outcomes: Although there was an average improvement in patient symptoms in the intervention group there was not enough evidence to suggest this was significant (CORE-OM), a difference in means of -0.35 (CI 0.52 to -1.22).<br>Satisfaction: Intervention practices had higher satisfaction levels<br>Voluntary sector usage: No difference in usage between groups
Marks (37)	Clinical outcomes: Intervention patients obtained significantly improved results than those on the wait list and under general practitioner care. There was also a high level of agreement in diagnosis and patient care management between nurse and a psychiatrist (used to assess if treatments were correct).
Bridges et al., (38)	Clinical Outcomes: Using ACORN, it was found that both Latinos and Whites had significant clinical improvement and there were no significant symptom improvement differences between them.<br>Service utilization: Non-Latinos have significantly increased post intervention telephone service utilization rates than Latinos. No difference in in-person visits.<br>Attendance of follow-up appointments: no significant differences in follow-up rates or attendance of appointments.<br>Satisfaction: Latino patients significantly more satisfied with BHC and first visits.
Friedli, King and Lloyd (39)	Clinical: There were no significant differences between the groups on any of the mental health outcomes at three or nine months. (BDI, BSI, CRCIS)<br>Costs: Direct and indirect counsellor costs are<br>£162 more per patient compared to GP care (3-months). However, the counsellor costs were £87 less per patient for the preceding 6 months. Overall counsellor group was more expensive.
Milne and Souter (40)	Clinical outcomes: There was a significant increase in active cognitive coping skills and a significant decrease in strain levels after 3 months (P<0.05).<br>Costs: Overall increase in drug costs over the period. The costs of psychotropic and other CNS drugs reduced in the improved coper group, whereas increased significantly in the other group. Reductions<br>in costs for the improved copers were due less drug usage, less hospital referrals and less GP visits
Magnée et al., (33)	Prescription effects: Although, there was an initial drop in prescription rates, mental health nurses in general practice did not decrease the overall prescription rates of antidepressants.
