# Supplementary material for: Clinical effectiveness and cost effectiveness of individual mental health workers colocated within primary care practices: a systematic literature review
Source: BMJ Open. 2020 Dec 2;10(12):e042052. doi: 10.1136/bmjopen-2020-042052 (PMC7713190; doi:10.1136/bmjopen-2020-042052)
Supplement: Author's manuscript [file bmjopen-2020-042052.draft_revisions.pdf]

BMJ Open

BMJ Open is committed to open peer review. As part of this commitment we make the peer review history of every article we publish publicly available.

When an article is published we post the peer reviewers' comments and the authors' responses online. We also post the versions of the paper that were used during peer review. These are the versions that the peer review comments apply to.

The versions of the paper that follow are the versions that were submitted during the peer review process. They are not the versions of record or the final published versions. They should not be cited or distributed as the published version of this manuscript.

BMJ Open is an open access journal and the full, final, typeset and author-corrected version of record of the manuscript is available on our site with no access controls, subscription charges or pay-per-view fees (http://bmjopen.bmj.com).

If you have any questions on BMJ Open's open peer review process please email info.bmjopen@bmj.com

BMJ Open

The clinical effectiveness and cost-effectiveness of individual mental health workers co-located within primary care practices

Journal:	*BMJ Open*
Manuscript ID	bmjopen-2020-042052
Article Type:	Original research
Date Submitted by the Author:	25-Jun-2020

Complete List of Authors:	Woods, Jean-Baptiste; Imperial College London, Department of Primary Care and Public Health, School of Public Health<br>Greenfield, Geva; Imperial College London, Department of Primary Care & Public Health<br>Majeed, Azeem; Imperial College, Primary Care<br>Hayhoe, Benedict; Imperial College London, Department of Primary Care and Public Health

Keywords:	PUBLIC HEALTH, PRIMARY CARE, MENTAL HEALTH

SCHOLARONE™
Manuscripts

BMJ

I, the Submitting Author has the right to grant and does grant on behalf of all authors of the Work (as defined in the below author licence), an exclusive licence and/or a non-exclusive licence for contributions from authors who are: i) UK Crown employees; ii) where BMJ has agreed a CC-BY licence shall apply, and/or iii) in accordance with the terms applicable for US Federal Government officers or employees acting as part of their official duties; on a worldwide, perpetual, irrevocable, royalty-free basis to BMJ Publishing Group Ltd ("BMJ") its licensees and where the relevant Journal is co-owned by BMJ to the co-owners of the Journal, to publish the Work in this journal and any other BMJ products and to exploit all rights, as set out in our licence.

The Submitting Author accepts and understands that any supply made under these terms is made by BMJ to the Submitting Author unless you are acting as an employee on behalf of your employer or a postgraduate student of an affiliated institution which is paying any applicable article publishing charge ("APC") for Open Access articles. Where the Submitting Author wishes to make the Work available on an Open Access basis (and intends to pay the relevant APC), the terms of reuse of such Open Access shall be governed by a Creative Commons licence – details of these licences and which Creative Commons licence will apply to this Work are set out in our licence referred to above.

Other than as permitted in any relevant BMJ Author's Self Archiving Policies, I confirm this Work has not been accepted for publication elsewhere, is not being considered for publication elsewhere and does not duplicate material already published. I confirm all authors consent to publication of this Work and authorise the granting of this licence.

For peer review only

The clinical effectiveness and cost-effectiveness of individual mental health workers co-located within primary care practices

Jean-Baptiste Woods[1], Geva Greenfield[1], Azeem Majeed,[1] Benedict WJ Hayhoe[1]

[1] Department of Primary Care and Public Health, School of Public Health, Imperial College London, UK

Corresponding author: Geva Greenfield, Department of Primary Care and Public Health, School of Public Health, Imperial College London, 314 The Reynolds Building, St Dunstan's Road, London W6 8RP, g.greenfield@imperial.ac.uk

Word count: Abstract 262, Main text 3213; **References:** 49; **Illustrations and tables**: In-text 2, Supplementary 7

Keywords: Primary health care; General Practice; Mental Health Services; Delivery of Health Care, Integrated; Delivery of Health Care

For peer review only

For peer review only - http://bmjopen.bmj.com/site/about/guidelines.xhtml

Ethics

No ethics approval was required for this study.

Authors contribution

JBW was involved with collecting the data, analysing the data, interpreting the data, and drafting the manuscript. GG contributed to the design, data collection, and revised the all drafts of the manuscript. BH conceived the idea for the study, contributed to the design and data collection, and revised all drafts of the manuscript. AM was involved in revising the manuscript. All authors read and approved the final manuscript.

Competing Interest: BH and AM are general practitioners working in the NHS.

We have not been prompted or paid by anyone to write this article.

For peer review only

Abstract

Background: Mental health disorders contribute significantly to the global burden of disease and lead to extensive strain on health system functionality. The integration of mental health workers into primary care has been proposed as one possible answer to this problem, but evidence of clinical and cost-effectiveness of this approach is currently unclear.

Objectives: To evaluate the clinical and cost-effectiveness of individual mental health workers co-located within primary care practices to deliver psychological treatments.

Design: Systematic literature review

Methodology: Quantitative studies identified through electronic database searches using MEDLINE, EMBASE, PsycINFO, HMIC, and Global Health. Two independent reviewers conducted title and abstract and full-text screening. Studies were also assessed for risk of bias.

Results: Fifteen studies from four countries were included. Mental health worker integration was associated with meaningful mental health benefits to varied populations, including minority groups and those with comorbid chronic disease. Furthermore, the intervention was correlated with high patient satisfaction and increases in specialist mental health referrals amongst minority populations. However, there was insufficient evidence to suggest whether clinical outcomes were significantly different from usual general practitioner care.

Conclusions: Whilst there appear to be some benefits associated with mental health worker integration in primary care practices, we found insufficient evidence to conclude that an onsite primary care mental health worker is significantly more clinically- or cost-effective when compared to usual general practitioner care. There should therefore be an increased emphasis on generating new evidence from clinical trials better to understand the benefits and effectiveness of mental health workers co-located in primary care practices.

For peer review only - http://bmjopen.bmj.com/site/about/guidelines.xhtml

Strengths and limitations of this study:

Strengths

- This review's inclusion of a broad range of population types enables it to be more representative of the general practice setting the review if focussed on.
- This review's inclusion of multiple different types of mental health worker can reduce the potential for the type of mental health worker being a confounding factor in whether significant outcome changes were identified.

Limitations

- Non-English language studies were not included in this review, therefore relevant evidence from non-English studies may have been missed.
- This study could have included a broader literature search that removed the database search terms relating specifically to integration, collocation and mental illnesses. This is hypothesised to be able to find slightly more relevant studies to review but will also increase the number of papers having to be screened exponentially.
- Pooled analysis within this review proved unfeasible, due to the relatively small number of studies identified and the heterogeneity of effects and outcomes investigated

For peer review only

Introduction

Mental health disorders contribute significantly to global disease incidence and prevalence, with one billion people affected by mental disorders(1) and 122.8 million disability-adjusted life years being lost annually through mental health problems.(2) The World Health Organization argues that primary care is the optimal environment to treat patients with these disorders,(3) but research has shown that primary care practitioners may be inadequately treating a substantial proportion of these (4,5). There have been many different quality improvement solutions envisioned in order to optimise the clinical and cost-effectiveness of mental health care within the primary care environment. A systematic review of 36 different interventions concluded that organisational interventions (e.g., integration or collaboration) improved the management of chronic conditions, whilst more simple interventions (e.g., practitioner education) were not effective (6).

One major proposed organisational intervention is the co-location or integration of mental health professionals within the primary care environment to deliver psychological therapies. This has achieved growing levels of traction due to the growing evidence base of the clinical (7,8) and cost-effectiveness (9–11) of psychological therapy and the increased patient satisfaction levels of this therapy compared to medication prescription (12). A recent focus of UK healthcare policy has been to try and implement this intervention within the NHS with the Government's Five-Year Forward View aiming to integrate 3,000 mental health therapists within primary care.(13) However, evidence for this policy is unclear: there are currently no systematic reviews specifically addressing whether the integration of mental health workers (MHW)s into primary care is clinically or cost-effective.

Previous systematic reviews have demonstrated improvement in clinical outcomes such as depression through mental health workers providing talking therapies (counselling and cognitive behavioural therapy) in primary care(8,14,15), although evidence for sustained improvements in social function and mental health is lacking.(16) There is a further lack of general consensus to suggest that cost-effectiveness and patient satisfaction levels are improved by this model(8,15). One review illustrated consultation rates and medication prescription rates decreased and referral rates increased significantly in the short-term, but not in the long term (17). This systematic review therefore aims to assess and appraise the evidence for the clinical effectiveness and cost-effectiveness of individual MHWs collocated within

For peer review only - http://bmjopen.bmj.com/site/about/guidelines.xhtml

primary care practices. This review also focuses on the individual worker effects caused by these mental health professionals rather than those created through collaborative care or consultation-liaison models. It is important to note that this review, through evaluating a broad range of mental health workers and patient populations, is expected to find a large degree of heterogeneity between the results of the papers identified. This is as a result of these studies having patients with varied symptomatic severity levels of and different prevalence of psychological disorders. Furthermore, different mental health workers may be more effective at treating certain disorders or reducing symptom prevalence/ severity which could further lead to heterogenous results.

Methods

This systematic review was carried out in accordance with the recommendations contained in the Cochrane Handbook for Systematic Reviews of Interventions (18) and reported in line with the PRISMA (Preferred Reporting Items for Systematic Reviews and Meta-Analyses) guidelines (19) (Supplement 5).

Eligibility Criteria

The PICOS (Population; Intervention; Comparison; Outcome; Study Design) framework was followed in identifying the eligibility criteria. All quantitative studies published before 1st July 2019 were eligible for the review. We included studies with participants of any age and gender. However, qualitative studies, studies with full text unavailable, and studies not in English were excluded. Systematic reviews were also excluded but were utilised for result comparisons.

Search strategy

The MEDLINE, EMBASE, PsycINFO, HMIC, and Global Health databases were searched via Ovid. The reference lists of related literature reviews were further searched for relevant sources. The search strategy was created by using MESH (Medical Subject Headings) and search terms related to four elements: 1) mental health disorders 2) the primary care setting, 3) MHWs, 4) co-location and integration. The Medline search can be found in Supplement 1; the full search strategy, for the other databases, is available from the authors on request.

For peer review only - http://bmjopen.bmj.com/site/about/guidelines.xhtml

Study selection

We used Covidence software (20) to select the final set of papers for the systematic review and remove duplicates. Title and abstract, and full-text screening were conducted by two independent reviewers (JBW and BH), with conflicts being resolved through meetings with a third researcher (GG).

Data extraction

Data were extracted using a standardized table which listed parameters including type of study design, type of primary care setting, type of MHW, age of participants, country of study implementation, sample size, and the main outcomes of the study.

Risk of Bias assessment

The Cochrane bias tool was used to assess the risk of bias of randomised trials whilst the Robins-I tool was used to assess the risk of bias of non-randomised trials(21,22). Studies were not excluded based on risk of bias.

Data analysis

Pooled analysis within this review proved unfeasible, due to the relatively small number of studies identified and the heterogeneity of effects and outcomes investigated, for example due to different models of integration or co-location.

Patient or Public involvement

It was not appropriate to involve patients or the public in the design, or conduct, or reporting, or dissemination plans of our research

For peer review only

Results

Study selection

Following removal of duplicates, 1863 titles and abstracts were screened to assess their suitability for inclusion within this review. Subsequently, 52 full-text papers were screened, of which 37 of these were deemed to be irrelevant. 15 papers were identified to be included (23–37) (Figure 1).

Study characteristics

Study characteristics are summarised within Table 1. Studies were predominantly conducted in the United Kingdom (25,27–29,32–34,36,37), with others being conducted in the Netherlands(26,30,31), the USA(23,35), and Canada (24). The majority of the papers fall either into the category of randomized controlled trial (five) (25,32–34,36) or observation descriptive studies (nine) (23,24,26,27,29–31,35,37). MHWs involved in the included studies were counsellors (24,25,28,36), mental health nurse specialists (26,30,31,34), clinical psychologists and (23,29,37) behavioural health consultants (35). Various forms of integration model were reported, ranging from a more replacement-structured integration model, which the majority of studies were based on, to a much more collaborative model, (23,35) with one paper assessing both the collaborative and replacement models of care(26).

For peer review only

Table 1: Characteristics of included studies

Author/ Date	Country of study	Study Design	Sample size	Primary care setting	Mental health worker
Kates et al., 2002 (24)	Canada	Observational descriptive study	3550 referrals	General practice	Counsellor
Cigrang˙ et al., 2006 (23)	United States of America	Observational descriptive study	234 primary care patients	Primary care clinics in a large military medical facility	Doctoral-level clinical psychologists and psychiatrist
Boot et al., 1994 (25)	United Kingdom	Randomised control trial	192 patients	General practice	Counsellors
Abidi et al., 2019 (26)	Netherlands	Observational descriptive study	155 practices	General practice	Practice mental health nurses and primary care psychologists
Evans et al., 2014 (27)	United Kingdom	Observational descriptive study	23 practices	General practice	Mental health link worker.
Spurgeon et al., 2005 (28)	United Kingdom	Controlled before-after study	271 patients	General practice	Counsellor
Pryde and Jachuck 1985 (29)	United Kingdom	Observational descriptive study	97 patients	General practice	Clinical psychologists

For peer review only - http://bmjopen.bmj.com/site/about/guidelines.xhtml

Magnée et al., 2016 (31)	Netherlands	Observational descriptive study	624,477 patients in 2010 1,392,187 patients in 2014.	General practice	Mental health nurses
McMahon et al., 2007 (32)	United Kingdom	Randomised controlled trial	62 patients	Primary care practices	Graduate mental health workers
Lester et al., (33)	United Kingdom	Cluster randomised controlled trial	368 patients	General practice	Mental health workers
Marks, 2007 (34)	United Kingdom	Randomised controlled trial	92 patients	General practice	Psychiatric nurse therapists
Bridges et al., 2014 (35)	United States of America	Observational descriptive study	793 patients	Primary care clinics	Behavioural health consultant
Friedli, King and Lloyd 2000 (36)	United Kingdom	Randomised controlled trial	136 patients	General Practice	Counsellor
Milne and Souter 1988 (37)	United Kingdom	Observational study	30 patients	General Practice	Clinical psychologist
Magnée et al., 2018 (30)	Netherlands	Observational Study	197,512	General Practice	Mental health Nurse

For peer review only - http://bmjopen.bmj.com/site/about/guidelines.xhtml

Quality assessment

We used two separate tools to carry out risk of bias assessment on all included studies (21,22). Most were of fair quality, with only a few indicators suggesting a high risk of bias (Supplements 2 and 3). Five papers were of fair quality(23,28,30,32,37), three of poor quality(25,34,36), two of unclear quality(27,29), five of good quality (24,26,31,33,35) (Supplements 6 and 7). However, risk of bias was generally higher within randomised controlled trials, which had a high-risk of performance bias. This is due to the nature of the trial as participants and personnel in all the RCTs were not able to be blinded as to the treatment they received. For non-randomised papers, missing data incurred the highest degree of bias. Quality assessment for included studies is available from the authors on request.

Outcomes

The study outcomes are summarized in Supplement 4.

Mental health outcomes

Eight papers reported a clinical benefit to integrating MHWs into primary care practices (23–25,28,29,34,35,37) compared to the usual care that a general practitioner would provide, whilst the remaining four papers did not report on these outcomes. For example, one study reported that symptoms of individuals in the integrated MHW program improved substantially, with average changes of 17.6 in CESD (Center for Epidemiologic Studies Depression Scale) score and 5.7 in GHQ (General Health Questionnaire) scores(24). Moreover, prior to the study commencing, individuals were assessed as reaching the threshold for inclusion based on CESD or GHQ scores. After treatment the total number of individuals exceeding threshold values had decreased significantly, 73% (CESD) and 82% (GHQ) ($P<0.005$). A paper by Cigrang et al., (2006) reported similar outcomes. OQ-45 scores decreased significantly for patients with more than one appointment. There was also a significant reduction in OQ-45 scores between patients that had either had 1, 2 or 4 or more appointments. This was also maintained during follow up (P range 0.032-0.001). However, three papers suggested that clinical improvements are not likely to be significantly greater than with usual GP care (32,33,36). One of these papers provides evidence that no significant differences, compared to standard care, for any of the mental health outcomes measures at either the three- or nine-month periods were able to be seen (36). There were three main scales found amongst the papers included, GHQ (24,25), BDI (32,36), CORE-OM (28,33). The other scales reported are OQ-45 (23), HADS (28), ACORN (35), CRCIS (36), SF-46 (28), CESD (24), HDRS17 (32), BSI (36), MADRS (32) and SASS

For peer review only - http://bmjopen.bmj.com/site/about/guidelines.xhtml

(32), although some papers included more than one scale and others did not report a scale at all.

Cost outcomes, psychotropic drug usage, and service utilization

In term of service utilization and referrals, there are seven papers that used these factors as one of their main evaluation outcomes (23–25,27,28,31,35). Only two papers provided comprehensive results data in terms of referrals(24,27), and these reporteddifferent findings. One (27) reported an increase in referrals to mental health specialists amongst minority communities and the other (24) reported a decrease in overall patient referrals. Service utilization rates varied between studies, with two reporting no difference in GP appointment rates between intervention and control groups(25,31). Conversely, another study showed a mean increase in GP appointments (6.8 to 8.4)(23) and another, investigating chronic conditions, showed a significantly reduced rate of health services utilization (GP appointments, home visits and patient referrals to mental health specialists) after 12-months(28). Two papers investigated the association between the intervention and the levels of psychotropic drug usage(25,30), and a further two papers evaluated the general costs associated with the intervention(36,37). Psychotropic drug usage was found to remain stable during and after the implementation of the intervention in one study(30), whilst another reported significantly fewer prescriptions as a result of the intervention(25). Finally, two studies evaluated the overall healthcare costs associated with the intervention(36,37). Both found increases in overall costs, which one attributed to increased prescribing costs(37). One study reported that MH worker intervention cost an additional £162 per patient per year compared with usual GP care.(36)

Patient satisfaction of the intervention

Five interventions were evaluated in terms of patient satisfaction levels (24,25,32,33,35). These papers illustrate a high level of satisfaction for this MHW integration and three of these papers further specify that this intervention was associated with significantly higher levels of patient satisfaction when compared to normal GP care(24,25,33). For example, Kates et al., (2002) illustrates that through using the CSQ scale consumers had an overall satisfaction of 92%, whilst Lester et al., (2007) using the same scale reports that intervention practices patients had higher mean levels of satisfaction than those in control practices ($P = 0.023$). The remaining two papers showed no significant differences in satisfaction levels between intervention and non-intervention patients (32,35).

For peer review only - http://bmjopen.bmj.com/site/about/guidelines.xhtml

Discussion

Summary

There is evidence that integration of MHWs in primary care practices provides meaningful mental health benefits to varied populations, including minority groups and those with comorbid chronic diseases. However, there is insufficient evidence to suggest that these improvements are clinically significant when compared with usual care. Similarly, the evidence base surrounding the cost-effectiveness of this intervention is mixed, with no common consensus as to whether integration of MHWs is more cost-effective than standard GP care. Service utilization and drug prescription rates vary considerably between studies, whilst referral rate changes suggest reductions in the overall burden on specialist care and improved access for minority groups. Patient satisfaction levels were consistently high in studies that had them as an outcome measure.

Comparison with existing literature

Mental health outcomes

The delivery of mental health treatments within the primary care environment has been consistently shown to be clinically effective (15,16,24,38), although this is most distinct within the short term. It is generally accepted that the integration of MHWs is likely to be correlated with decreased severity and a reduction of symptoms in patients with mental health problems. However, studies which included general care as a comparator provide no overall consensus as to whether there is any significant clinical benefit of integration over standard GP care. This finding may be as a result of standard GP care already having elements of GP-delivered counselling, which would therefore make significant changes in symptoms less likely as the treatment being delivered by the mental health worker would not be completely different.(38,39) Furthermore, the non-significant change in symptom reductions in certain studies may be attributed to the fact that the patients in these studies have less serious mental health problems; it would be unlikely for there to be a large effect size when integrating a mental health worker, as symptom levels and their severity are already low and so the scope for potential improvement is limited.

For peer review only - http://bmjopen.bmj.com/site/about/guidelines.xhtml

Cost outcomes, psychotropic drug usage and service utilization

Quality improvement strategies have generally been focussed primarily on reducing the burden of disease within the health care system where they are implemented (40–42). However, cost-containment has become necessary in recent years with healthcare systems worldwide being under strain from rising healthcare costs, recession and the burden of an aging population.(43–45) Integration of mental health practitioners has been devised as a solution to this problem as it is hypothesized that this could lead to reductions in resource utilization and in health service utilization, including fewer referrals to mental health specialists, fewer GP consultations and reduced drug prescriptions. However, this study and others assessing the overall resource utilization of the intervention suggest that it was in fact associated with increased cost.(8,15,36,37) There is a study that suggests that the intervention did not lead to increased costs, but this had a small sample size.(8)

One area where there is agreement between studies, concordant with the findings of this review, is in referral changes associated with MHW integration, with decreases in the frequency of referrals to mental health specialists well demonstrated in similar studies focussed on both specific MHWs (8,15) and MHWs in general.(17,46) If integrated mental health workers successfully reduce general practitioner appointment rates, this can allow GPs more time to treat other patients.(36) The results of one study found some evidence that referral to an MHW led to a small reduction in GP consultations, albeit with a small effect size.(17) This contrasts with the findings of our review, which finds no clear agreement between studies on whether integration of MHWs increased or decreased GP consultations.

Patient Satisfaction

In recent years the growing emphasis on and importance of patient and practitioner satisfaction has been frequently highlighted in terms of healthcare suitability and delivery.(47) Although satisfaction may not be a determinant indicator of success of an intervention,(48) satisfaction levels can ultimately decide whether utilization rates of the service are maintained and whether practitioners continue using a service over usual care. In accordance with the results of this review, patient satisfaction for MHW intervention in primary care has been shown to be consistently significantly higher than that of standard GP care.(15–17,46)

For peer review only - http://bmjopen.bmj.com/site/about/guidelines.xhtml

For peer review only

Limitations

We did not include non-English language studies, therefore relevant evidence from non-English studies may have been missed. This review has been unable to utilize a substantial number of papers related to the effectiveness, cost-effectiveness and other potential benefits of delivering specific psychological and psychosocial treatments within primary care. This is as a result of them not being focussed specifically on the effectiveness of the mental health worker itself. It was also difficult to include studies relating to collaborative care as it was not possible to determine whether the study's effects were owed to the MHW; some studies had more than one MHW within this collaborative care process which further increased uncertainty. Other studies were focussed on introducing certain interventions within primary care practices which were irrelevant to the review, including those of phone treatment, internet treatment and self-help. With more time and resource availability this study would have also included a broader literature search that removed the database search terms relating specifically to integration, collocation and mental illnesses. This is hypothesised to be able to find slightly more relevant studies to review but will also increase the number of papers having to be screened exponentially. The addition of mixed method studies and qualitative studies could have given a broader perspective and additional findings; however, this paper was solely interested in quantitative evaluations.

Implication for further research

Further research is needed to compare the clinical effectiveness of the integrated MHW with standard GP care with only five papers in this study comparing this. There also needs to be a greater emphasis on reporting what the usual care provided by primary care practitioners is, in order to accurately assess the clinical differences in care provided. Rigorous cost-effectiveness studies need to be created, which should assess cost-effectiveness to both the service provider and the broader health care system, and illustrate any societal costs associated with the intervention.

Implication for practice and policy

The World Health Organisation has advocated in recent years that the primary healthcare environment is the optimal environment for the treatment of a plethora of conditions, including mental health disorders(3). Thus, a multitude of structural interventions and changes have been proposed to fulfil this policy change effectively. One of the changes that has been proposed

For peer review only - http://bmjopen.bmj.com/site/about/guidelines.xhtml

and that this review has focussed on is that of the integration of an onsite mental health worker within primary care practices. This review has found that the integration of an MHW is correlated in some studies with decreased severity and a reduction of symptoms and therefore leads to better healthcare outcomes for patients with a range of mental health problems. This supports existing evidence that providing mental health care within the primary care setting is beneficial and that increasing resource expenditure on mental health workers will lead to beneficial impacts to the mental health of the populations exposed to them. However, there is insufficient evidence from this review to suggest that MHWs within primary care practices significantly elevate the quality of mental healthcare, compared to standard GP care. Likewise, there is little evidence to suggest that the intervention is more cost-effective, although this may be as a consequence of there being few studies examining this. Larger scale introductions of this intervention, and further randomised controlled trials, will enable more substantial and rigorous evaluations of clinical and cost-effectiveness. Until then, a degree of caution is needed in investing significant resources to implement this intervention. Furthermore, other possible policy and quality improvement recommendations relating to management of mental health problems should be investigated. For instance, a review has suggested that mental health treatments within secondary care may be more effective than those delivered in primary care, thus providing more resources to this environment may be more beneficial(49).

Conclusions

The NHS in England plans to increase the number of MHWs within primary care. This study suggests that further research is needed to evaluate both the clinical effectiveness and cost-effectiveness of this policy to ensure it is a good use of health system resources. Clarity is also needed on the best type of health professional to take on MHW roles within primary care.

Funding

This article presents independent research commissioned by the National Institute for Health Research (NIHR) under the Applied Health Research (ARC) programme for North West London. The views expressed in this publication are those of the author(s) and not necessarily those of the NHS, the NIHR or the Department of Health.

For peer review only - http://bmjopen.bmj.com/site/about/guidelines.xhtml

References

1. Rehm J, Shield KD. Global Burden of Disease and the Impact of Mental and Addictive Disorders. Curr Psychiatry Rep. 2019 Feb;21(2):10.

2. Global Burden Of Disease Results Tool, 2017 data [Internet]. Global Health Data Exchange. 2019 [cited 2019 Jul 13]. Available from: http://ghdx.healthdata.org/gbd-results-tool

3. World Health Organization, World Organisation of National Colleges, Academies and Academic Associations of General Practitioners/Family Physicians, editors. Integrating mental health into primary care: a global perspective. Geneva, Switzerland : London: World Health Organization ; Wonca; 2008. 206 p.

4. Nondetection of depression by primary care physicians reconsidered. - PubMed - NCBI [Internet]. [cited 2019 Jul 13]. Available from: https://www.ncbi.nlm.nih.gov/pubmed/7737492

5. Are There Detectable Differences in Quality of Care or Outcome of Depression across Primary Care Providers? on JSTOR [Internet]. [cited 2019 Jul 13]. Available from: https://www.jstor.org/stable/3767164

6. Gilbody S, Whitty P, Grimshaw J, Thomas R. Educational and Organizational Interventions to Improve the Management of Depression in Primary Care: A Systematic Review. JAMA. 2003 Jun 18;289(23):3145.

7. Høifødt RS, Strøm C, Kolstrup N, Eisemann M, Waterloo K. Effectiveness of cognitive behavioural therapy in primary health care: a review. Fam Pract. 2011 Oct;28(5):489–504.

8. Bower PJ, Rowland N. Effectiveness and cost effectiveness of counselling in primary care. In: The Cochrane Collaboration, editor. Cochrane Database of Systematic Reviews [Internet]. Chichester, UK: John Wiley & Sons, Ltd; 2006 [cited 2019 May 10]. p. CD001025.pub2. Available from: http://doi.wiley.com/10.1002/14651858.CD001025.pub2

9. Churchill R, Hunot V, Corney R, Knapp M, McGuire H, Tylee A, et al. A systematic review of controlled trials of the effectiveness and cost-effectiveness of brief psychological treatments for depression. Health Technol Assess Winch Engl. 2001;5(35):1–173.

10. Layard R. The case for psychological treatment centres. BMJ. 2006 Apr 27;332(7548):1030–2.

11. Laynard R, Clark D, Knapp M, Mayraz G. Cost-benefit analysis of psychological therapy. Natl Inst Econ Rev. 2007 Oct 1;202(1):90–8.

12. Priest RG, Vize C, Roberts A, Roberts M, Tylee A. Lay people's attitudes to treatment of depression: results of opinion poll for Defeat Depression Campaign just before its launch. BMJ. 1996 Oct 5;313(7061):858–9.

For peer review only

13. Cogan K, Akehurst J. Mental health nursing in the primary health care setting. Ment Health Nurs Dimens Prax 2nd Ed. 2014;(American Psychiatric Association. (2013). Diagnostic and statistical manual cimentai disorders (5th ed.). Arlington. American Psychiatric Association):335–53.

14. Hoifodt RS, Strom C, Kolstrup N, Eisemann M, Waterloo K. Effectiveness of cognitive behavioural therapy in primary health care: a review. Fam Pract. 2011 Oct 1;28(5):489–504.

15. Brettle A, Hill A, Jenkins P. Counselling in primary care: a systematic review of the evidence. Couns Psychother Res. 2008 Dec;8(4):207–14.

16. Bower P, Knowles S, Coventry PA, Rowland N. Counselling for mental health and psychosocial problems in primary care. Cochrane Common Mental Disorders Group, editor. Cochrane Database Syst Rev [Internet]. 2011 Sep 7 [cited 2019 Jun 26]; Available from: http://doi.wiley.com/10.1002/14651858.CD001025.pub3

17. Harkness E.F., Bower P.J. On-site mental health workers delivering psychological therapy and psychosocial interventions to patients in primary care: Effects on the professional practice of primary care providers. Cochrane Database Syst Rev. 2009;(1):CD000532.

18. Higgins JPT, Green S, Cochrane Collaboration, editors. Cochrane handbook for systematic reviews of interventions. Chichester, England ; Hoboken, NJ: Wiley-Blackwell; 2008. 649 p. (Cochrane book series).

19. Liberati A, Altman DG, Tetzlaff J, Mulrow C, Gotzsche PC, Ioannidis JPA, et al. The PRISMA statement for reporting systematic reviews and meta-analyses of studies that evaluate healthcare interventions: explanation and elaboration. BMJ. 2009 Dec 4;339(jul21 1):b2700–b2700.

20. Covidence. Covidence systematic review software [Internet]. Melbourne, Australia: Veritas Health Innovation; [cited 2019 Jul 28]. Available from: www.covidence.org

21. Sterne JA, Hernán MA, Reeves BC, Savović J, Berkman ND, Viswanathan M, et al. ROBINS-I: a tool for assessing risk of bias in non-randomised studies of interventions. BMJ. 2016 Oct 12;i4919.

22. Higgins JPT, Altman DG, Gotzsche PC, Juni P, Moher D, Oxman AD, et al. The Cochrane Collaboration's tool for assessing risk of bias in randomised trials. BMJ. 2011 Oct 18;343(oct18 2):d5928–d5928.

23. Cigrang JA, Dobmeyer AC, Becknell ME, Roa-Navarrete RA, Yerian SR. Evaluation of a collaborative mental health program in primary care: effects on patient distress and health care utilization. Prim Care Community Psychiatry. 2007 Feb 1;11(3):121–7.

24. Kates N, Crustolo A-M, Farrar S, Nikolaou L. Counsellors in Primary Care: Benefits and Lessons Learned. Can J Psychiatry. 2002 Nov;47(9):857–62.

25. Boot D, Gillies P, Fenelon J, Reubin R, Wilkins M, Gray P. Evaluation of the short-term impact of counseling in general practice. Patient Educ Couns. 1994 Aug;24(1):79–89.

For peer review only - http://bmjopen.bmj.com/site/about/guidelines.xhtml

26. Abidi L., Oenema A., Verhaak P., Tan F.E.S., van de Mheen D. The introduction of the practice nurse mental health in general practices in the Netherlands: effects on number of diagnoses of chronic and acute alcohol abuse. BMC Fam Pract. 2019;20(1):48.

27. Evans L, Green S, Sharma K. Improving access to primary mental health services : are link workers the answer? Lond J Prim Care. 2014;6:23–8.

28. Spurgeon P, Hicks C, Barwell F, Walton I, Spurgeon T. Counselling in primary care: A study of the psychological impact and cost benefits for four chronic conditions. Eur J Psychother Couns. 2005 Dec;7(4):269–90.

29. Pryde N.A., Jachuck S.J. Clinical psychologists in general practice: Data and observations. Public Health. 1985;99(2):89–97.

30. Magnee T., de Beurs D.P., Schellevis F.G., Verhaak P.F. Antidepressant prescriptions and mental health nurses: an observational study in Dutch general practice from 2011 to 2015. Scand J Prim Health Care. 2018;36(1):47–55.

31. Magnee T, de Beurs DP, de Bakker DH, Verhaak PF. Consultations in general practices with and without mental health nurses: an observational study from 2010 to 2014. BMJ Open. 2016;6(7):e011579.

32. McMahon L. Graduate mental health worker case management of depression in UK primary care : a pilot study. Br J Gen Pract. 2007;57(544):880–5.

33. Lester H, Freemantle N, Wilson S, Sorohan H, England E, Griffin C, et al. Cluster randomised controlled trial of the effectiveness of primary care mental health workers. Br J Gen Pract J R Coll Gen Pract. 2007 Mar;57(536):196–203.

34. Marks I. Controlled trial of psychiatric nurse therapists in primary care. BMJ. 1985 Apr 20;290(6476):1181–4.

35. Bridges AJ, Andrews ARI, Villalobos BT, Pastrana FA, Cavell TA, Gomez D. Does integrated behavioral health care reduce mental health disparities for Latinos? Initial findings. Aguilar-Gaxiola A Alegria, Alegria, Beck, Bernal, Blount, Borowsky, Bridges, Brown, Bryan, Cabassa, Corso, Dutton, Ennis, Escobar, Garces, Griner, Hilsenroth, Jones, Katon, Kessler, Kessler, Kouyoumdjian, Lagomasino, Lara, Miranda, Miranda, Mitchell, Olfson, Ortega, Ray-Sannerud, Roy-Byrne, Sanchez, Shattell, Sue, Sue, Tonigan, Vasquez, Vega, Voss Horrell, Wells, West, Young, editor. J Lat Psychol. 2014;2(1):37–53.

36. Friedli K, King MB, Lloyd M. The economics of employing a counsellor in general practice: analysis of data from a randomised controlled trial. Br J Gen Pract J R Coll Gen Pract. 2000 Apr;50(453):276–83.

37. Milne D, Souter K. A re-evaluation of the clinical psychologist in general practice. J R Coll Gen Pract. 1988 Oct;38(315):457–60.

38. Bortolotti B, Menchetti M, Bellini F, Montaguti MB, Berardi D. Psychological interventions for major depression in primary care: a meta-analytic review of randomized controlled trials. Gen Hosp Psychiatry. 2008 Jul;30(4):293–302.

For peer review only - http://bmjopen.bmj.com/site/about/guidelines.xhtml

39. Huibers M, Beurskens A, Bleijenberg G, Schayck CP van. Psychosocial interventions delivered by general practitioners. In: The Cochrane Collaboration, editor. Cochrane Database of Systematic Reviews [Internet]. Chichester, UK: John Wiley & Sons, Ltd; 2003 [cited 2019 Aug 12]. Available from: http://doi.wiley.com/10.1002/14651858.CD003494

40. Thompson C, Pulleyblank R, Parrott S, Essex H. The cost-effectiveness of quality improvement projects: a conceptual framework, checklist and online tool for considering the costs and consequences of implementation-based quality improvement: Cost-effectiveness of quality improvement. J Eval Clin Pract. 2016 Feb;22(1):26–30.

41. Severens JL. Value for money of changing healthcare services? Economic evaluation of quality improvement. Qual Saf Health Care. 2003 Oct 1;12(5):366–71.

42. Waters EA. Improving clinical effectiveness: a practical approach. J Eval Clin Pract. 1997 Nov;3(4):255–64.

43. Chandra A, Holmes J, Skinner J. Is This Time Different? The Slowdown in Health Care Spending. Brook Pap Econ Act. 2013;2013:261–302.

44. Lee R, Mason A. Cost of Aging. Finance Dev. 2017;54(1):7–9.

45. Stuckler D, Reeves A, Loopstra R, Karanikolos M, McKee M. Austerity and health: the impact in the UK and Europe. Eur J Public Health. 2017 Oct 1;27(suppl_4):18–21.

46. Bower P, Sibbald B. On-site mental health workers in primary care: effects on professional practice. Cochrane Database Syst Rev. 2000;(3):CD000532.

47. Berkowitz B. The Patient Experience and Patient Satisfaction: Measurement of a Complex Dynamic. Online J Issues Nurs. 2016 Jan 31;21(1):1.

48. Hemmings A. Counselling in primary care: A randomised controlled trial. Patient Educ Couns. 1997 Nov;32(3):219–30.

49. Raine R. Systematic review of mental health interventions for patients with common somatic symptoms: can research evidence from secondary care be extrapolated to primary care? BMJ. 2002 Nov 9;325(7372):1082–1082.

For peer review only

Figure legends:

Figure 1: PRISMA Flow diagram illustrating the selection process (19).

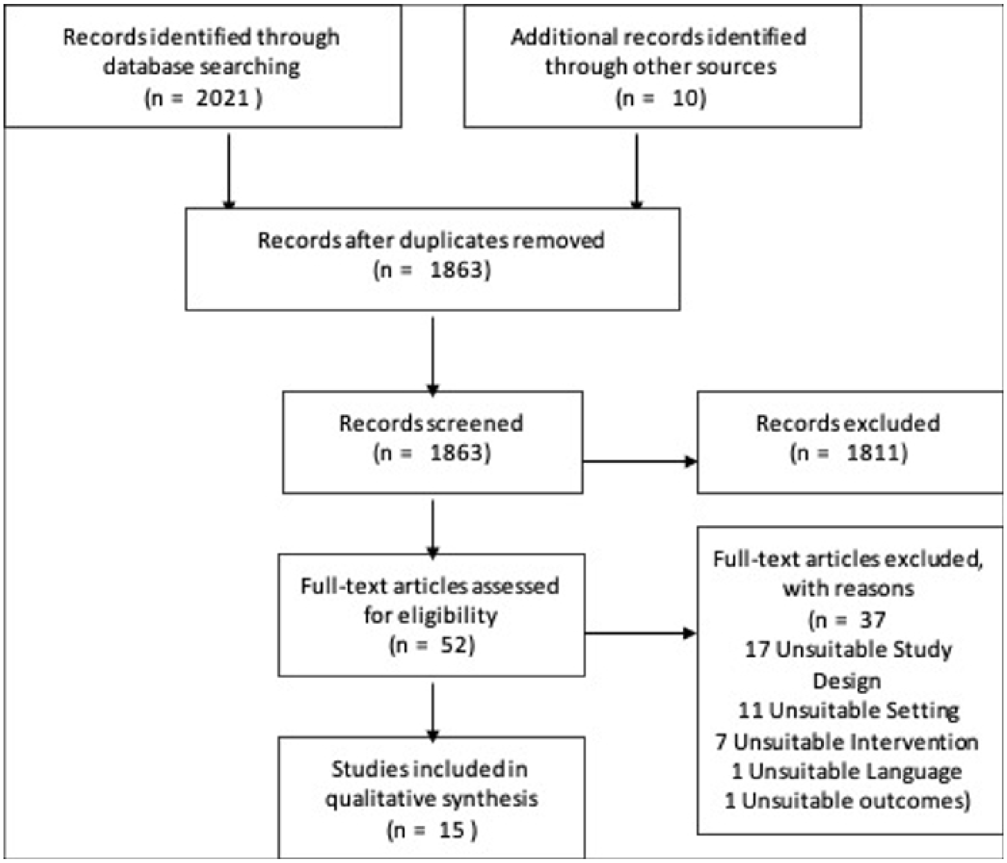

Figure 1: PRISMA Flow diagram illustrating the selection process (19).

Supplement 1: MEDLINE search strategy

1. exp Mental Disorders/ or mental disease*.mp. or exp Depression/ or mental disorder*.mp. or mental illness*.mp. or mental health illness*.mp.
2. mental disorder*.mp. [mp=title, abstract, original title, name of substance word, subject heading word, keyword heading word, protocol supplementary concept word, rare disease supplementary concept word, unique identifier]
3. Exp Mental Health/ or mental health problem*.mp. or exp Stress, Psychological/
4. stress.mp.
5. exp Depression/
6. depression.mp. [mp=title, abstract, original title, name of substance word, subject heading word, keyword heading word, protocol supplementary concept word, rare disease supplementary concept word, unique identifier]
7. exp Anxiety/ or anxiety.mp. or exp Anxiety Disorders/ or anxiety disorder*.mp.
8. (nervous breakdown$ or depression or depressive$ or affective disorder$ or mood disorder$ or anxiety disorder$ or phobia or phobic or panic disorder$ or PTSD or stress disorder$ or OCD or compulsive disorder$).mp. [mp=title, abstract, original title, name of substance word, subject heading word, keyword heading word, protocol supplementary concept word, rare disease supplementary concept word, unique identifier]
9. (anxi* or depress* or melancholi* or neuros* or neurotic or psychoneuro* or stress* or distress* or emotion*).mp.
10. ((mental$ or psychiatric or psychologic$) adj3 (illness$ or condition$ or disabil$ or disorder$ or disease$ or impair$ or problem)).mp. [mp=title, abstract, original title, name of substance word, subject heading word, keyword heading word, protocol supplementary concept word, rare disease supplementary concept word, unique identifier]
11. (bipolar or mania or dissociative disorder$ or neurosis or personality disorder$ or psychosis or paranoi$ or schizophren$ or schizoaffective disorder$ or psychotic).mp. [mp=title, abstract, original title, name of substance word, subject heading word, keyword heading word, protocol supplementary concept word, rare disease supplementary concept word, unique identifier]
12. 1 or 2 or 3 or 4 or 5 or 6 or 7 or 8 or 9 or 10 or 11
13. (Primary adj2 (care or health or healthcare)).mp. [mp=title, abstract, original title, name of substance word, subject heading word, keyword heading word, protocol supplementary concept word, rare disease supplementary concept word, unique identifier]
14. primary health care/ or *continuity of patient care*/ or patient-centred care or progressive patient care/
15. (Primary pratic* or Family pratic* or General practice*).mp. [mp=title, abstract, original title, name of substance word, subject heading word, keyword heading word, protocol supplementary concept word, rare disease supplementary concept word, unique identifier]
16. Community Health Services/
17. community Health Service*.mp. [mp=title, abstract, original title, name of substance word, subject heading word, keyword heading word, protocol supplementary concept word, rare disease supplementary concept word, unique identifier]
18. exp Community Mental Health Services/ or community care.mp. or Community Mental Health Service.mp. [mp=title, abstract, original title, name of substance word, subject heading word, keyword heading word, protocol supplementary concept word, rare disease supplementary concept word, unique identifier]
19. exp Family Practice/ or exp General Practice/
20. 13 or 14 or 15 or 16 or 17 or 18 or 19
21. (mental health adj2 (nurs* or therap* or counsellor* or worker* or practitioner*)).mp. [mp=title, abstract, original title, name of substance word, subject heading word, keyword heading word, protocol supplementary concept word, rare disease supplementary concept word, unique identifier]
22. psychotherapist.mp.
23. therapist*.mp.
24. counsellor*.mp.
25. 21 or 22 or 23 or 24

For peer review only

26. co-locat*.mp. [mp=title, abstract, original title, name of substance word, subject heading word, keyword heading word, protocol supplementary concept word, rare disease supplementary concept word, unique identifier]
27. colocat*.mp. [mp=title, abstract, original title, name of substance word, subject heading word, keyword heading word, protocol supplementary concept word, rare disease supplementary concept word, unique identifier]
28. integrat*.mp. [mp=title, abstract, original title, name of substance word, subject heading word, keyword heading word, protocol supplementary concept word, rare disease supplementary concept word, unique identifier]
29. ((integrat or within or collaborat* or colocat* or co-locat* or inside) adj primary health care).mp. [mp=title, abstract, original title, name of substance word, subject heading word, keyword heading word, protocol supplementary concept word, rare disease supplementary concept word, unique identifier]
30. ((integrat or within or collaborat* or colocat* or co-locat* or inside) adj primary care).mp. [mp=title, abstract, original title, name of substance word, subject heading word, keyword heading word, protocol supplementary concept word, rare disease supplementary concept word, unique identifier]
31. ((integrat or within or collaborat* or colocat* or co-locat* or inside) adj primary healthcare).mp. [mp=title, abstract, original title, name of substance word, subject heading word, keyword heading word, protocol supplementary concept word, rare disease supplementary concept word, unique identifier]
32. exp "Delivery of Health care, Integrated*/ or integrated care.mp. or integrated healthcare.mp. or integrated health care.mp.
33. collaborat*.mp. [mp=title, abstract, original title, name of substance word, subject heading word, keyword heading word, protocol supplementary concept word, rare disease supplementary concept word, unique identifier]
34. 26 or 27 or 28 or 29 or 30 or 31 or 32 or 33
35. 12 and 20 and 25 and 34

For peer review only

Supplement 2: Bias levels in non-randomised studies. Obtained via utilization of the Robins-I tool(15).

	Confounding bias	Bias in selection of participants	Bias in classification of interventions	Bias due to deviations from intended interventions	Bias due to missing data	Bias in measurement of outcomes	Bias in selection of the reported result
Kates et al., (24)	?	?	+	?	+	+	+
Cigrang et al., (23)	−	+	+	+	?	?	+
Abidi et al., (26)	?	+	+	+	?	+	+
Evans et al., (27)	?	?	?	+	?	+	+
Milne and Souter (37)	+	?	+	+	−	+	+
Bridges et al., (35)	+	+	+	+	−	−	+
Magnée et al., (31)	+	+	+	?	?	?	+
Pryde and Jachuck (29)	?	?	+	+	?	?	+
Spurgeon et al., (28)	+	−	?	+	−	+	+
Magnée et al., (30)	?	+	+	+	−	−	+

For peer review only

Supplement 3: Bias levels in randomised controlled trials. Obtained via utilization of the Cochrane bias tool(16).

	Random sequence generation	Allocation concealment	Blinding of participants and personnel	Blinding of outcome assessment	Incomplete outcome data	Selective reporting	Anything else, ideally prespecified
Boot et al., (25)	+	−	−	−	?	?	?
McMahon et al., (32)	?	?	−	+	−	+	?
Lester et al., (33)	+	+	−	?	+	+	?
Marks (34)	?	?	−	?	−	+	?
Friedli, King and Lloyd (36)	?	?	−	−	?	?	?

For peer review only

Supplement 4: Summary of findings in individual studies

Author	Findings
Kates et al., (24)	Clinical: An average change in score of 17.6 (CESD) and 5.7 (GHQ) was recorded. Furthermore, after comparing the numbers of patients classified as a case (due to exceeding cut off score) before and after treatment, there was 73% (CESD) and 82% (GHQ) less cases after treatment. All changes statistically significant ($P<0.005$).<br>Service utilization: 65% reduction in referrals to psychiatry outpatient since program started. Nonsignificant reduction of 10% in inpatient admissions.<br>Satisfaction: Overall consumer satisfaction rating of 92% (CSQ). Counsellors and practitioners satisfied.
Cigrang˙ et al., (23)	Clinical: OQ-45 scores decreased significantly for patients with more than one appointment. There was also a significant reduction in OQ-45 scores between patients that had either had 1, 2 or 4 or more appointments. This was also maintained during follow up (P range 0.032-0.001)<br>Service utilization: Significant increase in medical appointments $P<0.01$.
Boot et al., (25)	Clinical: Post intervention GHQ scores were significantly reduced for both the counselling intervention group and a control GP advice group. However, GHQ scores were significantly lower in the intervention group than in the control group and the average decrease in scores was twice as high in intervention group. There was a significantly larger proportion of the control group scoring above the GHQ threshold level post study, 46% compared to 25%.<br>Psychotropic drug usage:<br>Significantly fewer prescriptions during the 6- week period in the counselled group compared to GP care. Similar finding for only antidepressants but not anxiolytic drugs.<br>Referrals and consultation rates: Significantly higher referrals in GP group. No significant differences in follow-up consultations rates.<br>Satisfaction: significantly more counselled patients reported coping better with their problem, feeling happier, satisfied with treatment and had enough time to talk.
Abidi et al., (26)	Diagnoses: significant decrease of chronic alcohol abuse and acute alcohol abuse diagnoses for all groups (control, only PN-MH, only PCP, both PCP and PNMH). both PCP and PNMH higher mean of chronic and acute alcohol abuse diagnoses than control practices. No significant differences were found between groups.
Evans et al., (27)	Referrals: mean referral rate of BME patients for GP practices without a link worker 0.35 per week per 10000 patients and was unchanged throughout study. Referral rates for practices with a link worker increased from 0.65 to 1.37 referrals per week per 10000 patients.
Spurgeon et al., (28)	Clinical outcomes: All patients showed reductions in subjective distress (CORE-OM), excluding the diabetes group, and was maintained post-intervention. The HADS anxiety and depression scales show there was significant reductions in anxiety in all patient groups, patients with hypertension and frequent attenders had reductions in depression also. The total patient sample achieved significant reductions in the majority of SF36 subscales, excluding diabetic patients<br>Uptake of health services: Counselling intervention significantly reduced uptake of health services
Pryde and Jachuck (29)	Clinical outcomes: After an average of three consultations, out of 84 patients, 44% were considered to be much improved, 31% improved and 25% unchanged in terms of symptomatic severity and prevalence.
Magnée et al., (31)	Service utilization: GPs in practices with a mental health nurse have similar consultation rates than GPs without one. GPS with a mental health nurse have more patients exhibiting psychological or social disorders (OR=1.05). Nurses do not seem to replace GP care, but probably deliver additional consultations to patient

For peer review only

McMahon et al., (32)	Clinical outcomes: Little difference between the two treatments BDI. Although, significant gradual symptomatic improvement was seen over time for this measure. Some improvement over time was seen for other measures (HDRS17, MADRS and SASS) but was not significant and could be as a result of intervention group patients being significantly more likely to have medication adjustment (P<0.01).<br>Satisfaction: Patient satisfaction high for both treatments.
Lester et al., (33)	Clinical outcomes: Although there was an average improvement in patient symptoms in the intervention group there was not enough evidence to suggest this was significant (CORE-OM), a difference in means of -0.35 (CI 0.52 to -1.22).<br>Satisfaction: Intervention practices had higher satisfaction levels<br>Voluntary sector usage: No difference in usage between groups
Marks (34)	Clinical outcomes: Intervention patients obtained significantly improved results than those on the wait list and under general practitioner care. There was also a high level of agreement in diagnosis and patient care management between nurse and a psychiatrist (used to assess if treatments were correct).
Bridges et al., (35)	Clinical Outcomes: Using ACORN, it was found that both Latinos and Whites had significant clinical improvement and there were no significant symptom improvement differences between them.<br>Service utilization: Non-Latinos have significantly increased post intervention telephone service utilization rates than Latinos. No difference in in-person visits.<br>Attendance of follow-up appointments: no significant differences in follow-up rates or attendance of appointments.<br>Satisfaction: Latino patients significantly more satisfied with BHC and first visits.
Friedli, King and Lloyd (36)	Clinical: There were no significant differences between the groups on any of the mental health outcomes at three or nine months. (BDI, BSI, CRCIS)<br>Costs: Direct and indirect counsellor costs are<br>£162 more per patient compared to GP care (3-months). However, the counsellor costs were £87 less per patient for the preceding 6 months. Overall counsellor group was more expensive.
Milne and Souter (37)	Clinical outcomes: There was a significant increase in active cognitive coping skills and a significant decrease in strain levels after 3 months (P<0.05).<br>Costs: Overall increase in drug costs over the period. The costs of psychotropic and other CNS drugs reduced in the improved coper group, whereas increased significantly in the other group. Reductions<br>in costs for the improved copers were due less drug usage, less hospital referrals and less GP visits
Magnée et al., (30)	Prescription effects: Although, there was an initial drop in prescription rates, mental health nurses in general practice did not decrease the overall prescription rates of antidepressants.

For peer review only

Supplement 5: The PRISMA guidelines followed throughout this systematic review. The page numbers for each section are also included(13).

Section/topic	#	Checklist item	Reported on page #
**TITLE**			
Title	1	Identify the report as a systematic review, meta-analysis, or both.	1
**ABSTRACT**			
Structured summary:	2	Provide a structured summary including, as applicable: background; objectives; data sources; study eligibility criteria, participants, and interventions; study appraisal and synthesis methods; results; limitations; conclusions and implications of key findings; systematic review registration number.	3
**INTRODUCTION**			
Rationale:	3	Describe the rationale for the review in the context of what is already known.	5
Objectives:	4	Provide an explicit statement of questions being addressed with reference to participants, interventions, comparisons, outcomes, and study design (PICOS).	5
**METHODS**			
Protocol and registration	5	Indicate if a review protocol exists, if and where it can be accessed (e.g., Web address), and, if available, provide registration information including registration number.	-
Eligibility criteria	6	Specify study characteristics (e.g., PICOS, length of follow-up) and report characteristics (e.g., years considered, language, publication status) used as criteria for eligibility, giving rationale.	6
Information sources:	7	Describe all information sources (e.g., databases with dates of coverage, contact with study authors to identify additional studies) in the search and date last searched.	6
Search	8	Present full electronic search strategy for at least one database, including any limits used, such that it could be repeated.	6
Study selection	9	State the process for selecting studies (i.e., screening, eligibility, included in systematic review, and, if applicable, included in the meta-analysis).	7
Data collection process	10	Describe method of data extraction from reports (e.g., piloted forms, independently, in duplicate) and any processes for obtaining and confirming data from investigators.	7

For peer review only

Data items	11	List and define all variables for which data were sought (e.g., PICOS, funding sources) and any assumptions and simplifications made.	7
Risk of bias in individual studies	12	Describe methods used for assessing risk of bias of individual studies (including specification of whether this was done at the study or outcome level), and how this information is to be used in any data synthesis.	7
Summary measures	13	State the principal summary measures (e.g., risk ratio, difference in means).	-
Synthesis of results	14	Describe the methods of handling data and combining results of studies, if done, including measures of consistency (e.g., I^2) for each meta-analysis.	-

**Section/topic**	**#**	**Checklist item**	**Reported on page #**
Risk of bias across studies	15	Specify any assessment of risk of bias that may affect the cumulative evidence (e.g., publication bias, selective reporting within studies).	7
Additional analyses	16	Describe methods of additional analyses (e.g., sensitivity or subgroup analyses, meta-regression), if done, indicating which were pre-specified.	-
**RESULTS**			
Study selection	17	Give numbers of studies screened, assessed for eligibility, and included in the review, with reasons for exclusions at each stage, ideally with a flow diagram.	8
Study characteristics	18	For each study, present characteristics for which data were extracted (e.g., study size, PICOS, follow-up period) and provide the citations.	8
Risk of bias within studies	19	Present data on risk of bias of each study and, if available, any outcome level assessment (see item 12).	12
Results of individual studies	20	For all outcomes considered (benefits or harms), present, for each study: (a) simple summary data for each intervention group (b) effect estimates and confidence intervals, ideally with a forest plot.	12
Synthesis of results	21	Present results of each meta-analysis done, including confidence intervals and measures of consistency.	-
Risk of bias across studies	22	Present results of any assessment of risk of bias across studies (see Item 15).	12
Additional analysis	23	Give results of additional analyses, if done (e.g., sensitivity or subgroup analyses, meta-regression [see Item 16]).	-
**DISCUSSION**			

For peer review only

Summary of evidence	24	Summarize the main findings including the strength of evidence for each main outcome; consider their relevance to key groups (e.g., healthcare providers, users, and policy makers).	14
Limitations	25	Discuss limitations at study and outcome level (e.g., risk of bias), and at review-level (e.g., incomplete retrieval of identified research, reporting bias).	16
Conclusions	26	Provide a general interpretation of the results in the context of other evidence, and implications for future research.	17
**FUNDING**			
Funding	27	Describe sources of funding for the systematic review and other support (e.g., supply of data); role of funders for the systematic review.	-

For peer review only

Supplement 6: Bias assessment for randomised controlled trials, summary of results.

Cochrane Risk of Bias Tool for Randomized Controlled Trials.

Author	Selection bias		Performance bias	Detection bias	Attrition bias	Reporting bias	Other bias	Total
	Random sequence generation	Allocation concealment	Blinding of participants and personnel	Blinding of outcome assessment	Incomplete outcome data	Selective reporting	Anything else, ideally prespecified	
Boot et al., (25)	Low	High	High	High	Unclear	Unclear	Unclear	Poor quality
McMahon et al., (32)	Unclear	Unclear	High	Low	High	Low	Unclear	Fair quality
Lester et al., (33)	Low	Low	High	Unclear	Low	Low	Unclear	Good quality
Marks (34)	Unclear	Unclear	High	Unclear	High	Low	Unclear	Poor quality
Friedli, King and Lloyd (36)	Unclear	Unclear	High	High	Unclear	Unclear	Unclear	Poor quality

For peer review only

Supplement 7: Bias assessment in non-randomised studies of interventions, summary of results. Risk Of Bias In Non-randomized Studies of Interventions (ROBINS-I) tool.

Author	Pre-intervention		At intervention	Post intervention				Overall bias
	Confounding bias	Bias in selection of participants	Bias in classification of interventions	Bias due to deviations from intended interventions	Bias due to missing data	Bias in measurement of outcomes	Bias in selection of the reported result	
Kates et al., (24)	Unclear	Unclear	Low	Unclear	Low	Low	Low	Good Quality
Cigrang et al., (23)	High	Low	Low	Low	Unclear	Unclear	Low	Fair Quality
Abidi et al., (26)	Unclear	Low	Low	Low	Unclear	Low	Low	Good Quality
Evans et al., (27)	Unclear	Unclear	Uncertain	Low	Unclear	Low	Low	Unclear Quality
Milne and Souter (37)	Low	Unclear	Low	Low	High	Low	Low	Fair quality
Bridges et al., (35)	Low	Low	Low	Low	High	High	Low	Good quality
Magnée et al., (31)	Low	Low	Low	Unclear	Unclear	Unclear	Low	Good quality
Pryde and Jachuck (29)	Unclear	Unclear	Low	Low	Unclear	Unclear	Low	Unclear quality
Spurgeon et al., (28)	Low	High	Unclear	Low	High	Low	Low	Fair Quality
Magnée et al., (30)	Unclear	Low	Low	Low	High	High	Low	Fair Quality

For peer review only

BMJ Open

The clinical effectiveness and cost-effectiveness of individual mental health workers co-located within primary care practices. A systematic literature review.

Journal:	*BMJ Open*
Manuscript ID	bmjopen-2020-042052.R1
Article Type:	Original research
Date Submitted by the Author:	28-Sep-2020
Complete List of Authors:	Woods, Jean-Baptiste; Imperial College London, Department of Primary Care and Public Health, School of Public Health<br>Greenfield, Geva; Imperial College London, Department of Primary Care & Public Health<br>Majeed, Azeem; Imperial College London, Primary Care<br>Hayhoe, Benedict; Imperial College London, Department of Primary Care and Public Health
**Primary Subject Heading**:	General practice / Family practice
Secondary Subject Heading:	Mental health, Public health
Keywords:	PUBLIC HEALTH, PRIMARY CARE, MENTAL HEALTH

SCHOLARONE™
Manuscripts

I, the Submitting Author has the right to grant and does grant on behalf of all authors of the Work (as defined in the below author licence), an exclusive licence and/or a non-exclusive licence for contributions from authors who are: i) UK Crown employees; ii) where BMJ has agreed a CC-BY licence shall apply, and/or iii) in accordance with the terms applicable for US Federal Government officers or employees acting as part of their official duties; on a worldwide, perpetual, irrevocable, royalty-free basis to BMJ Publishing Group Ltd ("BMJ") its licensees and where the relevant Journal is co-owned by BMJ to the co-owners of the Journal, to publish the Work in this journal and any other BMJ products and to exploit all rights, as set out in our licence.

The Submitting Author accepts and understands that any supply made under these terms is made by BMJ to the Submitting Author unless you are acting as an employee on behalf of your employer or a postgraduate student of an affiliated institution which is paying any applicable article publishing charge ("APC") for Open Access articles. Where the Submitting Author wishes to make the Work available on an Open Access basis (and intends to pay the relevant APC), the terms of reuse of such Open Access shall be governed by a Creative Commons licence – details of these licences and which Creative Commons licence will apply to this Work are set out in our licence referred to above.

Other than as permitted in any relevant BMJ Author's Self Archiving Policies, I confirm this Work has not been accepted for publication elsewhere, is not being considered for publication elsewhere and does not duplicate material already published. I confirm all authors consent to publication of this Work and authorise the granting of this licence.

For peer review only

The clinical effectiveness and cost-effectiveness of individual mental health workers co-located within primary care practices. A systematic literature review.

Jean-Baptiste Woods[1], Geva Greenfield[1], Azeem Majeed,[1] Benedict WJ Hayhoe[1]

[1] Department of Primary Care and Public Health, School of Public Health, Imperial College London, UK

Corresponding author: Geva Greenfield, Department of Primary Care and Public Health, School of Public Health, Imperial College London, 314 The Reynolds Building, St Dunstan's Road, London W6 8RP, g.greenfield@imperial.ac.uk

Word count: Abstract 299, Main text 3673; **References:** 52; **Illustrations and tables**: In-text 2, Supplementary 8

Keywords: Primary health care; General Practice; Mental Health Services; Delivery of Health Care, Integrated; Delivery of Health Care

For peer review only

Ethics

No ethics approval was required for this study.

Authors contribution

JBW was involved with collecting the data, analysing the data, interpreting the data, and drafting the manuscript. GG contributed to the design, data collection, and revised the all drafts of the manuscript. BH conceived the idea for the study, contributed to the design and data collection, and revised all drafts of the manuscript. AM was involved in revising the manuscript. All authors read and approved the final manuscript.

For peer review only

Abstract

Objectives: Mental health disorders contribute significantly to the global burden of disease and lead to extensive strain on health systems. The integration of mental health workers into primary care has been proposed as one possible solution, but evidence of clinical and cost-effectiveness of this approach is unclear. We reviewed the clinical and cost-effectiveness of mental health workers co-located within primary care practices.

Design: Systematic literature review

Data Sources: We searched the MEDLINE, EMBASE, PsycINFO, HMIC, and Global Health databases

Eligibility Criteria: All quantitative studies published before July 2019 were eligible for the review, participants of any age and gender were included. Studies did not need to report a certain outcome measure or comparator in order to be eligible.

Data Extraction and Synthesis: Data were extracted using a standardized table, however, pooled analysis proved unfeasible. Studies were assessed for risk of bias utilising the ROBINS-I-Tool and the Cochrane Collaboration's tool for assessing risk of bias in randomised trials.

Results: Fifteen studies from four countries were included. Mental health worker integration was associated with mental health benefits to varied populations, including minority groups and those with comorbid chronic diseases. Furthermore, the interventions were correlated with high patient satisfaction and increases in specialist mental health referrals amongst minority populations. However, there was insufficient evidence to suggest clinical outcomes were significantly different from usual general practitioner care.

Conclusions: Whilst there appear to be some benefits associated with mental health worker integration in primary care practices, we found insufficient evidence to conclude that an onsite primary care mental health worker is significantly more clinically- or cost-effective when compared to usual general practitioner care. There should therefore be an increased emphasis on generating new evidence from clinical trials to better understand the benefits and effectiveness of mental health workers co-located within primary care practices.

For peer review only - http://bmjopen.bmj.com/site/about/guidelines.xhtml

For peer review only

Strengths and limitations of this study:

Strengths

- This review's inclusion of a broad range of population types enables it to be more representative of the general practice setting the review is focussed on.
- This review's inclusion of multiple different types of mental health worker can reduce the potential for the type of mental health worker being a confounding factor in whether significant outcome changes were identified.

Limitations

- Non-English language studies were not included in this review, therefore relevant evidence from non-English studies may have been missed.
- This study could have included a broader literature search that removed the database search terms relating specifically to integration, collocation and mental illnesses. This is hypothesised to be able to find slightly more relevant studies to review but will also increase the number of papers having to be screened exponentially.
- Pooled analysis within this review proved unfeasible, due to the relatively small number of studies identified and the heterogeneity of effects and outcomes investigated

For peer review only

Introduction

Mental health disorders contribute significantly to global disease incidence and prevalence, with one billion people affected by mental disorders(1) and 122.8 million disability-adjusted life years being lost annually through mental health problems (2). The World Health Organization argues that primary care is the optimal environment to treat patients with these disorders (3), but research has shown that primary care practitioners may be inadequately treating a substantial proportion of these (4,5). There have been many different quality improvement solutions envisioned in order to optimise the clinical and cost-effectiveness of mental health care within the primary care environment. A systematic review of 36 different interventions concluded that organisational interventions (e.g., integration or collaboration) improved the management of chronic mental health conditions, whilst more simple interventions (e.g., practitioner education) were not effective (6).

One major proposed organisational intervention is the co-location or integration of mental health professionals within the primary care environment to deliver psychological therapies. The co-location of mental health professionals within primarily practices refers to the location of these professionals within the same offices/ clinical space as the primary care practice staff. These professionals are full members of the primary healthcare team receiving both self-referrals from patients but also referrals from all other team members, which can include GPs, clinical pharmacists, practice nurses and Health Care Assistants (7). These professionals would generally also be expected to attend meetings within their assigned practice/ clinics, and liaise with clinicians across other mental health, social care and physical health (7). This intervention has achieved increasing levels of traction due to the growing evidence base of the clinical (8,9) and cost-effectiveness (10–12) of psychological therapy and the increased patient satisfaction levels of this therapy compared to medication prescription (13). A recent focus of UK healthcare policy has been to try and implement this intervention within the NHS with the Government's Five-Year Forward View aiming to integrate 3,000 mental health therapists within primary care (14,15). According to the most recent figures, outlined within Health Education England's workforce strategy (December 2017), there are over 2,130 more mental health therapists (83.7%) employed in England (16). Around 800 of these therapists were subsequently co-located into general practice (16). However, evidence for this policy is unclear: there are currently no systematic reviews

For peer review only - http://bmjopen.bmj.com/site/about/guidelines.xhtml

specifically addressing whether the integration of mental health workers (MHW)s into primary care is clinically or cost-effective.

Previous systematic reviews have demonstrated improvement in clinical outcomes such as depression through mental health workers providing talking therapies (counselling and cognitive behavioural therapy) in primary care(9,17,18), although evidence for sustained improvements in social function and mental health is lacking.(19) There is a further lack of general consensus to suggest that cost-effectiveness and patient satisfaction levels are improved by this model(9,18). One review illustrated consultation rates and medication prescription rates decreased and referral rates increased significantly in the short-term, but not in the long term (20). This systematic review therefore aims to assess and appraise the evidence for the clinical effectiveness and cost-effectiveness of individual MHWs co-located within primary care practices. This review also focuses on the individual worker effects caused by these mental health professionals rather than those created through collaborative care or consultation-liaison models. It is important to note that this review, through evaluating a broad range of mental health workers and patient populations, is expected to find a large degree of heterogeneity between the results of the papers identified. This is as a result of these studies having patients with varied symptomatic severity levels of, and different prevalence of, psychological disorders. Furthermore, different mental health workers may be more effective at treating certain disorders or reducing symptom prevalence/ severity which could further lead to heterogenous results.

Methods

This systematic review was carried out in accordance with the recommendations contained in the Cochrane Handbook for Systematic Reviews of Interventions (21) and reported in line with the PRISMA (Preferred Reporting Items for Systematic Reviews and Meta-Analyses) guidelines (22) (Supplement 1). The search was conducted in July 2019.

Eligibility Criteria

The PICOS (Population; Intervention; Comparison; Outcome; Study Design) framework was followed in identifying the eligibility criteria. All quantitative studies published before 1st July 2019 were eligible for the review, including randomised controlled trials (RCTs), practice-based evidence studies, and cost-effectiveness studies. We included studies with participants of any age and gender. Qualitative studies, studies with full text unavailable, and studies not in

For peer review only - http://bmjopen.bmj.com/site/about/guidelines.xhtml

English were excluded. Systematic reviews were also excluded but were utilised for result comparisons. Studies did not need to report a certain outcome measure in order to be eligible for inclusion (i.e. GHQ, BDI, or CORE-OM). It was also preferable to have studies which included standard general practitioner care as a comparator, but as studies with this were limited in number it was not used to restrict the eligibility criteria.

Search strategy

The MEDLINE, EMBASE, PsycINFO, HMIC, and Global Health databases were searched via Ovid. The reference lists of related literature reviews were further searched for relevant sources. The search strategy was created by using MESH (Medical Subject Headings) and search terms related to four elements: 1) mental health disorders 2) the primary care setting, 3) MHWs, 4) co-location and integration. The Medline search can be found in Supplement 2; the full search strategy, for the other databases, is available from the authors on request.

Study selection

We used Covidence software (23) to select the final set of papers for the systematic review and remove duplicates. Title and abstract, and full-text screening were conducted by two independent reviewers (JBW and BH), with conflicts being resolved through meetings with a third researcher (GG).

Data extraction

Data were extracted using a standardized table (table 1) which listed parameters including type of study design, type of primary care setting, type of MHW, age of participants, country of study implementation, sample size, and the main outcomes of the study.

Risk of Bias assessment

The Cochrane bias tool was used to assess the risk of bias of randomised trials whilst the Robins-I tool was used to assess the risk of bias of non-randomised trials(24,25). Studies were not excluded based on risk of bias.

For peer review only - http://bmjopen.bmj.com/site/about/guidelines.xhtml

Data analysis

Pooled analysis within this review proved unfeasible, due to the relatively small number of studies identified and the heterogeneity of effects and outcomes investigated, for example due to different models of integration or co-location.

Patient or Public involvement

It was not appropriate to involve patients or the public in the design, or conduct, or reporting, or dissemination plans of our research

Results

Study selection

Following removal of duplicates, 1863 titles and abstracts were screened to assess their suitability for inclusion within this review. Subsequently, 52 full-text papers were screened, of which 37 were deemed to be irrelevant. 15 papers were identified to be included (26–40) (Figure 1).

Study characteristics

Study characteristics are summarised within Table 1. Studies were predominantly conducted in the United Kingdom (28,30–32,35–37,39,40), with others being conducted in the Netherlands(29,33,34), the USA(26,38), and Canada (27). The majority of the papers fall either into the category of randomized controlled trial (five) (28,35–37,39) or observation descriptive studies (nine) (26,27,29,30,32–34,38,40). MHWs involved in the included studies were counsellors (27,28,31,39), mental health nurse specialists (29,33,34,37), clinical psychologists and (26,32,40) behavioural health consultants (38). Various forms of integration model were reported, ranging from a more replacement-structured integration model, which the majority of studies were based on, to a much more collaborative model, (26,38) with one paper assessing both the collaborative and replacement models of care(29).

For peer review only

Table 1: Characteristics of included studies

Author/ Date	Country of study	Study Design	Sample size	Age of participants	Primary care setting	Mental health worker
Kates et al., 2002 (27)	Canada	Observational descriptive study	3550 patients	No age limitation/ range not reported	General practice	Counsellor
Cigrang˙ et al., 2006 (26)	United States of America	Observational descriptive study	234 patients	18 to 87 years	Primary care clinics in a large military medical facility	Doctoral-level clinical psychologists and psychiatrist
Boot et al., 1994 (28)	United Kingdom	Randomised control trial	192 patients	16 years or above	General practice	Counsellors
Abidi et al., 2019 (29)	Netherlands	Observational descriptive study	~15,000 patients	No age limitation/ range not reported	General practice	Practice mental health nurses and primary care psychologists
Evans et al., 2014 (30)	United Kingdom	Observational descriptive study	65,000 patients	No age limitation/ range not reported	General practice	Mental health link worker.
Spurgeon et al., 2005 (31)	United Kingdom	Controlled before-after study	271 patients	No age limitation/ range not reported	General practice	Counsellor
Pryde and Jachuck 1985 (32)	United Kingdom	Observational descriptive study	97 patients	14 to 74 years	General practice	Clinical psychologists

For peer review only - http://bmjopen.bmj.com/site/about/guidelines.xhtml

Magnée et al., 2016 (34)	Netherlands	Observational descriptive study	624,477 patients in 2010 1,392,187 patients in 2014.	No age limitation/ range not reported	General practice	Mental health nurses
McMahon et al., 2007 (35)	United Kingdom	Randomised controlled trial	62 patients	18–65 years	Primary care practices	Graduate mental health workers
Lester et al., 2007 (36)	United Kingdom	Cluster randomised controlled trial	368 patients	18–65 years	General practice	Mental health workers
Marks, 1985 (37)	United Kingdom	Randomised controlled trial	92 patients	No age limitation/ range not reported	General practice	Psychiatric nurse therapists
Bridges et al., 2014 (38)	United States of America	Observational descriptive study	793 patients	1 to 75 years	Primary care clinics	Behavioural health consultant
Friedli, King and Lloyd 2000 (39)	United Kingdom	Randomised controlled trial	136 patients	No age limitation/ range not reported	General Practice	Counsellor
Milne and Souter 1988 (40)	United Kingdom	Observational descriptive study	30 patients	No age limitation/ range not reported	General Practice	Clinical psychologist
Magnée et al., 2018 (33)	Netherlands	Observational descriptive study	197,512 patients	10 to 65 years	General Practice	Mental health Nurse

For peer review only - http://bmjopen.bmj.com/site/about/guidelines.xhtml

Quality assessment

We used two separate tools to carry out risk of bias assessment on all included studies (24,25). Most were of fair quality, with only a few indicators suggesting a high risk of bias (Supplements 3 and 4). Five papers were of fair quality(26,31,33,35,40), three of poor quality(28,37,39), two of unclear quality(30,32), five of good quality (27,29,34,36,38) (Supplements 5 and 6). However, risk of bias was generally higher within randomised controlled trials, which had a high-risk of performance bias. This is due to the nature of the intervention as participants and personnel in all the RCTs were not able to be blinded as to the treatment they received. For non-randomised papers, missing data incurred the highest degree of bias. Quality assessment for included studies is available from the authors on request.

Outcomes

The study outcomes are summarized in Supplement 7.

Mental health outcomes

There were three main scales found amongst the papers included, GHQ (27,28), BDI (35,39), CORE-OM (31,36). The other scales reported are OQ-45 (26), HADS (31), ACORN (38), CRCIS (39), SF-46 (31), CESD (27), HDRS17 (35), BSI (39), MADRS (35) and SASS (35), although some papers included more than one scale and others did not report a scale at all. Eight papers reported a clinical benefit to integrating MHWs into primary care practices (26–28,31,32,37,38,40) compared to the usual care that a general practitioner would provide, whilst four papers did not report on these outcomes(29,30,33,34). For example, one study reported that symptoms of individuals in the integrated MHW program improved substantially, with average changes of 17.6 in CESD (Center for Epidemiologic Studies Depression Scale) score and 5.7 in GHQ (General Health Questionnaire) scores(27). Moreover, prior to the study commencing, individuals were assessed as reaching the threshold for inclusion based on CESD or GHQ scores. After treatment the total number of individuals exceeding threshold values had decreased significantly, 73% (CESD) and 82% (GHQ) ($P<0.005$). A paper by Cigrang et al., (2006) reported similar outcomes. OQ-45 scores decreased significantly for patients with more than one appointment. There was also a significant reduction in OQ-45 scores between patients that had either had 1, 2 or 4 or more appointments. This was also maintained during follow up (P range 0.032-0.001). However, three papers suggested that clinical improvements are not likely to be significantly greater than with usual GP care (35,36,39). One of these papers provides evidence that no significant differences, compared to standard care, for any of the

For peer review only - http://bmjopen.bmj.com/site/about/guidelines.xhtml

mental health outcomes measures at either the three- or nine-month periods were able to be seen (39).

Cost outcomes, psychotropic drug usage, and service utilization

In terms of service utilization and referrals, there are seven papers that used these factors as one of their main evaluation outcomes (26–28,30,31,34,38). Only two papers provided comprehensive results data in terms of referrals(27,30), and these reported different findings. One (30) reported an increase in referrals to mental health specialists amongst minority communities and the other (27) reported a decrease in overall patient referrals. Service utilization rates varied between studies, with two reporting no difference in GP appointment rates between intervention and control groups(28,34). Conversely, another study showed a mean increase in GP appointments (6.8 to 8.4)(26) and another, investigating chronic conditions, showed a significantly reduced rate of health services utilization (GP appointments, home visits and patient referrals to mental health specialists) after 12-months(31). Two papers investigated the association between the intervention and the levels of psychotropic drug usage(28,33), and a further two papers evaluated the general costs associated with the intervention(39,40). Psychotropic drug usage was found to remain stable during and after the implementation of the intervention in one study(33), whilst another reported significantly fewer prescriptions as a result of the intervention(28). Finally, there were only two studies identified from the search that providing data evaluating the cost-effectiveness of the intervention(39,40). Both of these studies found increases in overall costs. One study reported that the MHW intervention cost an additional £162 per patient per year (indirectly and directly) compared with usual GP care for the first three months, although in the preceding 6 months costs were £87 less per patient (39). The other study reported overall increases in drug costs over the period, however the results varied between groups within the study. The costs of psychotropic and other drugs reduced in the improved coper group (11 individuals), whereas they increased significantly in the remaining 11 individuals which accounted for the overall group trend. Reductions in costs for the improved copers were due less drug usage, less hospital referrals and less GP visits.

For peer review only - http://bmjopen.bmj.com/site/about/guidelines.xhtml

Patient satisfaction of the intervention

Five interventions were evaluated in terms of patient satisfaction levels (27,28,35,36,38). These papers illustrate a high level of satisfaction for this MHW integration and three of these papers further specify that this intervention was associated with significantly higher levels of patient satisfaction when compared to normal GP care(27,28,36). For example, Kates et al., (2002) illustrates that through using the CSQ scale consumers had an overall satisfaction of 92%, whilst Lester et al., (2007) using the same scale reports that intervention practices patients had higher mean levels of satisfaction than those in control practices (P = 0.023). The remaining two papers showed no significant differences in satisfaction levels between intervention and non-intervention patients (35,38).

Discussion

Summary

There is evidence from this review that the integration of MHWs in primary care practices provides meaningful mental health benefits to varied populations, including minority groups and those with comorbid chronic diseases (26–28,31,32,37,38,40). However, there is insufficient evidence to suggest that these improvements are clinically significant when compared with usual care. Similarly, the evidence base surrounding the cost-effectiveness of this intervention is mixed, with no common consensus as to whether integration of MHWs is more cost-effective than standard GP care. Service utilization and drug prescription rates vary considerably between studies, whilst referral rate changes suggest reductions in the overall burden on specialist care and improved access for minority groups. Patient satisfaction levels were consistently high in studies that measured this construct.

Comparison with existing literature

Mental health outcomes

The delivery of mental health treatments within the primary care environment has been consistently shown to be clinically effective (18,19,27,41), although this is most distinct within the short term (one to six months). Primary care policy supporting integration of MHWs in primary care practices suggests an expectation that the integration of MHW's is likely to be correlated with decreased severity and a reduction of symptoms in patients with mental health

For peer review only - http://bmjopen.bmj.com/site/about/guidelines.xhtml
For peer review only

problems. However, studies which included general care as a comparator provide no overall consensus as to whether there is any significant clinical benefit of integration over standard GP care. This finding may be as a result of standard GP care already having elements of GP-delivered counselling. This would make significant changes in symptoms less likely, as the treatment being delivered by the mental health worker would not be completely different (41,42). Furthermore, the non-significant change in symptom reductions in certain studies may be attributed to the fact that the patients in these studies have less serious mental health problems. Consequently, it would be unlikely for there to be a large effect size when integrating a mental health worker, as symptom levels and their severity are already low or moderate and so the scope for potential improvement is limited. For instance, in the paper by McMahon et al., (2007) the baseline characteristics of the patients for numerous mental health outcome scales were usually rated as moderate, BDI 26.2 (moderate depression), HDRS17 18.1 (20 or higher is classed as moderate severity), MADRS 24.3 (moderate depression). Moreover, it is possible that the small effect size of integrating MHWs within primary care practices was as a consequence of the included primary care practices being self-selecting. Therefore, the practices included would have had an established interest in mental health service provision, and as a consequence were more likely to be participants in the included studies, whilst also being expected to already perform well in terms of general mental health care.

Cost outcomes, psychotropic drug usage and service utilization

Quality improvement strategies have generally been focussed primarily on reducing the burden of disease within the health care system where they are implemented (43–45). However, cost-containment has become necessary in recent years with healthcare systems worldwide being under strain from rising healthcare costs, recession and the burden of an aging population.(46–48) Integration of mental health practitioners has been devised as a solution to this problem as it is hypothesized that this could lead to reductions in resource utilization and in health service utilization, including fewer referrals to mental health specialists, fewer GP consultations and reduced drug prescriptions. However, this study and others assessing the overall resource utilization of the intervention suggest that it is in fact associated with increased cost.(9,18,39,40) There is a study that suggests that the intervention does not lead to increased costs, but this had a small sample size.(9)

One area where there is agreement between studies, concordant with the findings of this review, is in referral changes associated with MHW integration. Decreases in the frequency

For peer review only - http://bmjopen.bmj.com/site/about/guidelines.xhtml

of referrals to mental health specialists are well demonstrated in similar studies focussed on both specific MHWs (9,18) and MHWs in general(20,49). If integrated mental health workers successfully reduce general practitioner appointment rates, this can allow GPs more time to treat other patients(39). The results of one study found some evidence that referral to an MHW led to a small reduction in GP consultations, albeit with a small effect size.(20) This contrasts with the findings of our review, which finds no clear agreement between studies on whether integration of MHWs increased or decreased GP consultations.

Patient Satisfaction

In recent years the growing emphasis on and importance of patient and practitioner satisfaction has been frequently highlighted in terms of healthcare suitability and delivery.(50) Although satisfaction may not be a determinant indicator of success of an intervention,(51) satisfaction levels can ultimately decide whether utilization rates of the service are maintained and whether practitioners continue using a service over usual care. In accordance with the results of this review, patient satisfaction for MHW intervention in primary care has been shown to be consistently significantly higher than that of standard GP care.(18–20,49)

Limitations

We did not include non-English language studies, therefore relevant evidence from non-English studies may have been missed. This review has been unable to utilize a substantial number of papers related to the effectiveness, cost-effectiveness and other potential benefits of delivering specific psychological and psychosocial treatments within primary care. This is as a result of them not being focussed specifically on the effectiveness of the mental health worker itself. It was also difficult to include studies relating to collaborative care as it was not possible to determine whether the study's effects were owed to the MHW; some studies had more than one MHW within this collaborative care process which further increased uncertainty. Other studies were focussed on introducing certain interventions within primary care practices which were irrelevant to the review, including those of phone treatment, internet treatment and self-help. With more time and resource availability this study would have also included a broader literature search that removed the database search terms relating specifically to integration, collocation and mental illnesses. This is hypothesised to be able to find slightly more relevant studies to review but will also increase the number of papers having to be screened exponentially. The addition of mixed method studies and qualitative

For peer review only - http://bmjopen.bmj.com/site/about/guidelines.xhtml

studies could have given a broader perspective and additional findings; however, this paper was solely interested in quantitative evaluations.

Implication for further research

Further research is needed to compare the clinical effectiveness of the integrated MHW with standard GP care with only five papers in this study comparing this. There also needs to be a greater emphasis on reporting what the usual care provided by primary care practitioners is, in order to accurately assess the clinical differences in care provided. Rigorous cost-effectiveness studies need to be created, which should assess cost-effectiveness to both the service provider and the broader health care system, and illustrate any societal costs associated with the intervention.

Implication for practice and policy

The World Health Organisation has advocated in recent years that the primary healthcare environment is the optimal environment for the treatment of a plethora of conditions, including mental health disorders(3). Thus, a multitude of structural interventions and changes have been proposed to fulfil this policy change effectively. One of the changes that has been proposed and that this review has focussed on is that of the integration of an onsite mental health worker within primary care practices. This review has found that the integration of an MHW is correlated in some studies with decreased severity and a reduction of symptoms and therefore leads to better healthcare outcomes for patients with a range of mental health problems. This supports existing evidence that providing mental health care within the primary care setting is beneficial and that increasing resource expenditure on mental health workers will lead to beneficial impacts to the mental health of the populations exposed to them. However, there is insufficient evidence from this review to suggest that MHWs within primary care practices significantly elevate the quality of mental healthcare, compared to standard GP care. Likewise, there is little evidence to suggest that the intervention is more cost-effective, although this may be as a consequence of there being few studies examining this. Larger scale introductions of this intervention, and further randomised controlled trials, will enable more substantial and rigorous evaluations of clinical and cost-effectiveness. Until then, a degree of caution is needed in investing significant resources to implement this intervention. Furthermore, other possible policy and quality improvement recommendations relating to management of mental health problems should be investigated. For instance, a review has suggested that mental health

For peer review only

treatments within secondary care may be more effective than those delivered in primary care, thus providing more resources to this environment may be more beneficial(52).

Conclusions

The NHS in England plans to increase the number of MHWs within primary care. This study suggests that further research is needed to evaluate both the clinical effectiveness and cost-effectiveness of this policy to ensure it is a good use of health system resources. Clarity is also needed on the best type of health professional to take on MHW roles within primary care.

Funding

This article presents independent research commissioned by the National Institute for Health Research (NIHR) under the Applied Health Research (ARC) programme for North West London. The views expressed in this publication are those of the author(s) and not necessarily those of the NHS, the NIHR or the Department of Health.

Competing Interest

BH and AM are general practitioners working in the NHS.

We have not been prompted or paid by anyone to write this article.

Data Sharing Statement

All papers and data in this review can be found utilising the submitted search strategy. All papers can be found through OVID. The search strategies for the listed databases are available upon reasonable request, as is the quality assessment data.

For peer review only

References

1. Rehm J, Shield KD. Global Burden of Disease and the Impact of Mental and Addictive Disorders. Curr Psychiatry Rep. 2019 Feb;21(2):10.

2. Global Burden Of Disease Results Tool, 2017 data [Internet]. Global Health Data Exchange. 2019 [cited 2019 Jul 13]. Available from: http://ghdx.healthdata.org/gbd-results-tool

3. World Health Organization, World Organisation of National Colleges, Academies and Academic Associations of General Practitioners/Family Physicians, editors. Integrating mental health into primary care: a global perspective. Geneva, Switzerland : London: World Health Organization ; Wonca; 2008. 206 p.

4. Nondetection of depression by primary care physicians reconsidered. - PubMed - NCBI [Internet]. [cited 2019 Jul 13]. Available from: https://www.ncbi.nlm.nih.gov/pubmed/7737492

5. Are There Detectable Differences in Quality of Care or Outcome of Depression across Primary Care Providers? on JSTOR [Internet]. [cited 2019 Jul 13]. Available from: https://www.jstor.org/stable/3767164

6. Gilbody S, Whitty P, Grimshaw J, Thomas R. Educational and Organizational Interventions to Improve the Management of Depression in Primary Care: A Systematic Review. JAMA. 2003 Jun 18;289(23):3145.

7. NHS England/Mental Health/Improving Access to Psychological Therapies. NHSE guidance-co-locating-mental-health-therapists-primary-care.pdf [Internet]. NHS England; 2018 [cited 2019 Jul 22]. Available from: https://www.england.nhs.uk/publication/guidance-on-co-locating-mental-health-therapists-in-primary-care/

8. Høifødt RS, Strøm C, Kolstrup N, Eisemann M, Waterloo K. Effectiveness of cognitive behavioural therapy in primary health care: a review. Fam Pract. 2011 Oct;28(5):489–504.

9. Bower PJ, Rowland N. Effectiveness and cost effectiveness of counselling in primary care. In: The Cochrane Collaboration, editor. Cochrane Database of Systematic Reviews [Internet]. Chichester, UK: John Wiley & Sons, Ltd; 2006 [cited 2019 May 10]. p. CD001025.pub2. Available from: http://doi.wiley.com/10.1002/14651858.CD001025.pub2

10. Churchill R, Hunot V, Corney R, Knapp M, McGuire H, Tylee A, et al. A systematic review of controlled trials of the effectiveness and cost-effectiveness of brief psychological treatments for depression. Health Technol Assess Winch Engl. 2001;5(35):1–173.

11. Layard R. The case for psychological treatment centres. BMJ. 2006 Apr 27;332(7548):1030–2.

12. Laynard R, Clark D, Knapp M, Mayraz G. Cost-benefit analysis of psychological therapy. Natl Inst Econ Rev. 2007 Oct 1;202(1):90–8.

For peer review only - http://bmjopen.bmj.com/site/about/guidelines.xhtml

13. Priest RG, Vize C, Roberts A, Roberts M, Tylee A. Lay people's attitudes to treatment of depression: results of opinion poll for Defeat Depression Campaign just before its launch. BMJ. 1996 Oct 5;313(7061):858–9.

14. NHS England. NHS England » Implementing the Five Year Forward View for Mental Health [Internet]. [cited 2020 Sep 15]. Available from: https://www.england.nhs.uk/publication/implementing-the-fyfv-for-mental-health/

15. Stepping forward to 2020/21: The mental health workforce plan for England [Internet]. BASW. 2017 [cited 2020 Sep 15]. Available from: https://www.basw.co.uk/resources/stepping-forward-202021-mental-health-workforce-plan-england

16. Health Education England. Workforce strategy: A draft health and care workforce strategy for England to 2027 [Internet]. Health Education England. 2017 [cited 2020 Sep 16]. Available from: https://www.hee.nhs.uk/our-work/workforce-strategy

17. Hoifodt RS, Strom C, Kolstrup N, Eisemann M, Waterloo K. Effectiveness of cognitive behavioural therapy in primary health care: a review. Fam Pract. 2011 Oct 1;28(5):489–504.

18. Brettle A, Hill A, Jenkins P. Counselling in primary care: a systematic review of the evidence. Couns Psychother Res. 2008 Dec;8(4):207–14.

19. Bower P, Knowles S, Coventry PA, Rowland N. Counselling for mental health and psychosocial problems in primary care. Cochrane Common Mental Disorders Group, editor. Cochrane Database Syst Rev [Internet]. 2011 Sep 7 [cited 2019 Jun 26]; Available from: http://doi.wiley.com/10.1002/14651858.CD001025.pub3

20. Harkness E.F., Bower P.J. On-site mental health workers delivering psychological therapy and psychosocial interventions to patients in primary care: Effects on the professional practice of primary care providers. Cochrane Database Syst Rev. 2009;(1):CD000532.

21. Higgins JPT, Green S, Cochrane Collaboration, editors. Cochrane handbook for systematic reviews of interventions. Chichester, England ; Hoboken, NJ: Wiley-Blackwell; 2008. 649 p. (Cochrane book series).

22. Liberati A, Altman DG, Tetzlaff J, Mulrow C, Gotzsche PC, Ioannidis JPA, et al. The PRISMA statement for reporting systematic reviews and meta-analyses of studies that evaluate healthcare interventions: explanation and elaboration. BMJ. 2009 Dec 4;339(jul21 1):b2700–b2700.

23. Covidence. Covidence systematic review software [Internet]. Melbourne, Australia: Veritas Health Innovation; [cited 2019 Jul 28]. Available from: www.covidence.org

24. Sterne JA, Hernán MA, Reeves BC, Savović J, Berkman ND, Viswanathan M, et al. ROBINS-I: a tool for assessing risk of bias in non-randomised studies of interventions. BMJ. 2016 Oct 12;i4919.

For peer review only

25. Higgins JPT, Altman DG, Gotzsche PC, Juni P, Moher D, Oxman AD, et al. The Cochrane Collaboration's tool for assessing risk of bias in randomised trials. BMJ. 2011 Oct 18;343(oct18 2):d5928–d5928.

26. Cigrang JA, Dobmeyer AC, Becknell ME, Roa-Navarrete RA, Yerian SR. Evaluation of a collaborative mental health program in primary care: effects on patient distress and health care utilization. Prim Care Community Psychiatry. 2007 Feb 1;11(3):121–7.

27. Kates N, Crustolo A-M, Farrar S, Nikolaou L. Counsellors in Primary Care: Benefits and Lessons Learned. Can J Psychiatry. 2002 Nov;47(9):857–62.

28. Boot D, Gillies P, Fenelon J, Reubin R, Wilkins M, Gray P. Evaluation of the short-term impact of counseling in general practice. Patient Educ Couns. 1994 Aug;24(1):79–89.

29. Abidi L., Oenema A., Verhaak P., Tan F.E.S., van de Mheen D. The introduction of the practice nurse mental health in general practices in the Netherlands: effects on number of diagnoses of chronic and acute alcohol abuse. BMC Fam Pract. 2019;20(1):48.

30. Evans L, Green S, Sharma K. Improving access to primary mental health services : are link workers the answer? Lond J Prim Care. 2014;6:23–8.

31. Spurgeon P, Hicks C, Barwell F, Walton I, Spurgeon T. Counselling in primary care: A study of the psychological impact and cost benefits for four chronic conditions. Eur J Psychother Couns. 2005 Dec;7(4):269–90.

32. Pryde N.A., Jachuck S.J. Clinical psychologists in general practice: Data and observations. Public Health. 1985;99(2):89–97.

33. Magnee T., de Beurs D.P., Schellevis F.G., Verhaak P.F. Antidepressant prescriptions and mental health nurses: an observational study in Dutch general practice from 2011 to 2015. Scand J Prim Health Care. 2018;36(1):47–55.

34. Magnee T, de Beurs DP, de Bakker DH, Verhaak PF. Consultations in general practices with and without mental health nurses: an observational study from 2010 to 2014. BMJ Open. 2016;6(7):e011579.

35. McMahon L. Graduate mental health worker case management of depression in UK primary care : a pilot study. Br J Gen Pract. 2007;57(544):880–5.

36. Lester H, Freemantle N, Wilson S, Sorohan H, England E, Griffin C, et al. Cluster randomised controlled trial of the effectiveness of primary care mental health workers. Br J Gen Pract J R Coll Gen Pract. 2007 Mar;57(536):196–203.

37. Marks I. Controlled trial of psychiatric nurse therapists in primary care. BMJ. 1985 Apr 20;290(6476):1181–4.

38. Bridges AJ, Andrews ARI, Villalobos BT, Pastrana FA, Cavell TA, Gomez D. Does integrated behavioral health care reduce mental health disparities for Latinos? Initial findings. Aguilar-Gaxiola A Alegria, Alegria, Beck, Bernal, Blount, Borowsky, Bridges, Brown, Bryan, Cabassa, Corso, Dutton, Ennis, Escobar, Garces, Griner, Hilsenroth, Jones, Katon, Kessler, Kessler, Kouyoumdjian, Lagomasino, Lara, Miranda, Miranda, Mitchell, Olfson, Ortega, Ray-Sannerud, Roy-Byrne, Sanchez, Shattell, Sue, Sue,

For peer review only

For peer review only - http://bmjopen.bmj.com/site/about/guidelines.xhtml

Tonigan, Vasquez, Vega, Voss Horrell, Wells, West, Young, editor. J Lat Psychol. 2014;2(1):37–53.

39. Friedli K, King MB, Lloyd M. The economics of employing a counsellor in general practice: analysis of data from a randomised controlled trial. Br J Gen Pract J R Coll Gen Pract. 2000 Apr;50(453):276–83.

40. Milne D, Souter K. A re-evaluation of the clinical psychologist in general practice. J R Coll Gen Pract. 1988 Oct;38(315):457–60.

41. Bortolotti B, Menchetti M, Bellini F, Montaguti MB, Berardi D. Psychological interventions for major depression in primary care: a meta-analytic review of randomized controlled trials. Gen Hosp Psychiatry. 2008 Jul;30(4):293–302.

42. Huibers M, Beurskens A, Bleijenberg G, Schayck CP van. Psychosocial interventions delivered by general practitioners. In: The Cochrane Collaboration, editor. Cochrane Database of Systematic Reviews [Internet]. Chichester, UK: John Wiley & Sons, Ltd; 2003 [cited 2019 Aug 12]. Available from: http://doi.wiley.com/10.1002/14651858.CD003494

43. Thompson C, Pulleyblank R, Parrott S, Essex H. The cost-effectiveness of quality improvement projects: a conceptual framework, checklist and online tool for considering the costs and consequences of implementation-based quality improvement: Cost-effectiveness of quality improvement. J Eval Clin Pract. 2016 Feb;22(1):26–30.

44. Severens JL. Value for money of changing healthcare services? Economic evaluation of quality improvement. Qual Saf Health Care. 2003 Oct 1;12(5):366–71.

45. Waters EA. Improving clinical effectiveness: a practical approach. J Eval Clin Pract. 1997 Nov;3(4):255–64.

46. Chandra A, Holmes J, Skinner J. Is This Time Different? The Slowdown in Health Care Spending. Brook Pap Econ Act. 2013;2013:261–302.

47. Lee R, Mason A. Cost of Aging. Finance Dev. 2017;54(1):7–9.

48. Stuckler D, Reeves A, Loopstra R, Karanikolos M, McKee M. Austerity and health: the impact in the UK and Europe. Eur J Public Health. 2017 Oct 1;27(suppl_4):18–21.

49. Bower P, Sibbald B. On-site mental health workers in primary care: effects on professional practice. Cochrane Database Syst Rev. 2000;(3):CD000532.

50. Berkowitz B. The Patient Experience and Patient Satisfaction: Measurement of a Complex Dynamic. Online J Issues Nurs. 2016 Jan 31;21(1):1.

51. Hemmings A. Counselling in primary care: A randomised controlled trial. Patient Educ Couns. 1997 Nov;32(3):219–30.

52. Raine R. Systematic review of mental health interventions for patients with common somatic symptoms: can research evidence from secondary care be extrapolated to primary care? BMJ. 2002 Nov 9;325(7372):1082–1082.

For peer review only

Figure legends:

Figure 1: PRISMA Flow diagram illustrating the selection process (22).

For peer review only

For peer review only - http://bmjopen.bmj.com/site/about/guidelines.xhtml

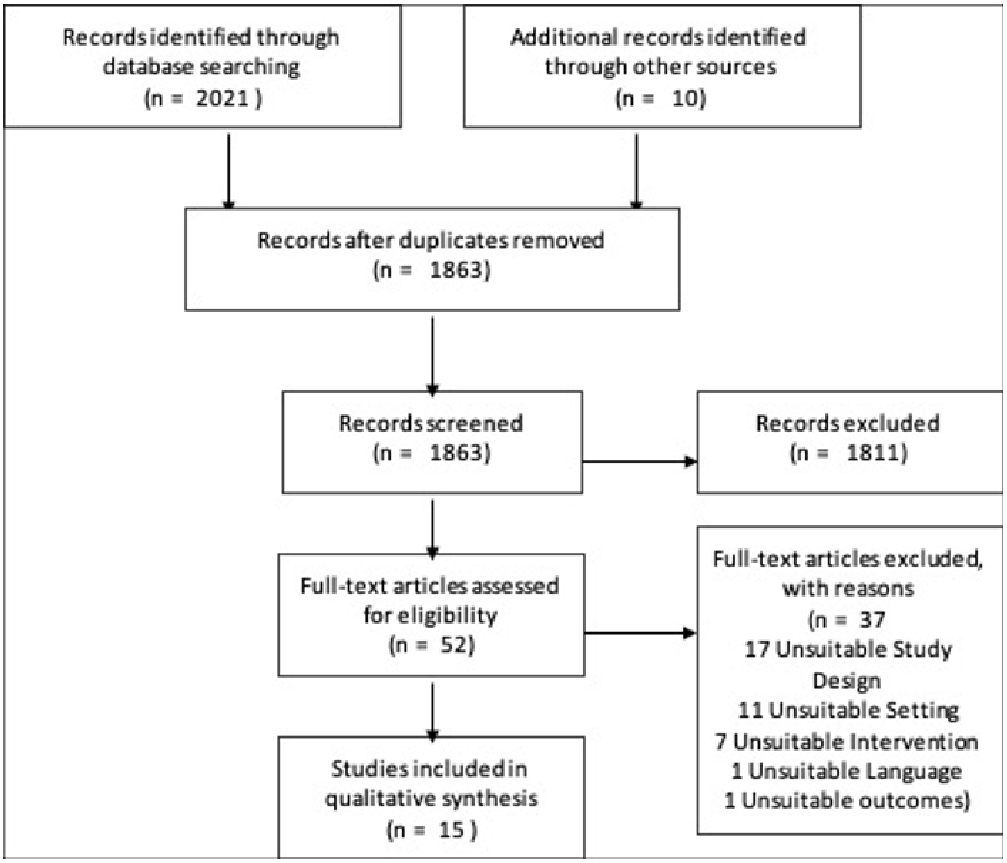

Figure 1: PRISMA Flow diagram illustrating the selection process (19).

Supplement 1': The PRISMA guidelines followed throughout this systematic review. The page numbers for each section are also included(13).

Section/topic	#	Checklist item	Reported on page #
**TITLE**			
Title	1	Identify the report as a systematic review, meta-analysis, or both.	1
**ABSTRACT**			
Structured summary:	2	Provide a structured summary including, as applicable: background; objectives; data sources; study eligibility criteria, participants, and interventions; study appraisal and synthesis methods; results; limitations; conclusions and implications of key findings; systematic review registration number.	3
**INTRODUCTION**			
Rationale:	3	Describe the rationale for the review in the context of what is already known.	5
Objectives:	4	Provide an explicit statement of questions being addressed with reference to participants, interventions, comparisons, outcomes, and study design (PICOS).	6
**METHODS**			
Protocol and registration	5	Indicate if a review protocol exists, if and where it can be accessed (e.g., Web address), and, if available, provide registration information including registration number.	-
Eligibility criteria	6	Specify study characteristics (e.g., PICOS, length of follow-up) and report characteristics (e.g., years considered, language, publication status) used as criteria for eligibility, giving rationale.	6
Information sources:	7	Describe all information sources (e.g., databases with dates of coverage, contact with study authors to identify additional studies) in the search and date last searched.	6
Search	8	Present full electronic search strategy for at least one database, including any limits used, such that it could be repeated.	7
Study selection	9	State the process for selecting studies (i.e., screening, eligibility, included in systematic review, and, if applicable, included in the meta-analysis).	7
Data collection process	10	Describe method of data extraction from reports (e.g., piloted forms, independently, in duplicate) and any processes for obtaining and confirming data from investigators.	7

For peer review only

Data items	11	List and define all variables for which data were sought (e.g., PICOS, funding sources) and any assumptions and simplifications made.	7
Risk of bias in individual studies	12	Describe methods used for assessing risk of bias of individual studies (including specification of whether this was done at the study or outcome level), and how this information is to be used in any data synthesis.	7
Summary measures	13	State the principal summary measures (e.g., risk ratio, difference in means).	-
Synthesis of results	14	Describe the methods of handling data and combining results of studies, if done, including measures of consistency (e.g., I^2) for each meta-analysis.	-

For peer review only

Section/topic	#	Checklist item	Reported on page #
Risk of bias across studies	15	Specify any assessment of risk of bias that may affect the cumulative evidence (e.g., publication bias, selective reporting within studies).	7
Additional analyses	16	Describe methods of additional analyses (e.g., sensitivity or subgroup analyses, meta-regression), if done, indicating which were pre-specified.	-
**RESULTS**			
Study selection	17	Give numbers of studies screened, assessed for eligibility, and included in the review, with reasons for exclusions at each stage, ideally with a flow diagram.	8
Study characteristics	18	For each study, present characteristics for which data were extracted (e.g., study size, PICOS, follow-up period) and provide the citations.	8
Risk of bias within studies	19	Present data on risk of bias of each study and, if available, any outcome level assessment (see item 12).	11
Results of individual studies	20	For all outcomes considered (benefits or harms), present, for each study: (a) simple summary data for each intervention group (b) effect estimates and confidence intervals, ideally with a forest plot.	11
Synthesis of results	21	Present results of each meta-analysis done, including confidence intervals and measures of consistency.	-
Risk of bias across studies	22	Present results of any assessment of risk of bias across studies (see Item 15).	11
Additional analysis	23	Give results of additional analyses, if done (e.g., sensitivity or subgroup analyses, meta-regression [see Item 16]).	-
**DISCUSSION**			

Summary of evidence	24	Summarize the main findings including the strength of evidence for each main outcome; consider their relevance to key groups (e.g., healthcare providers, users, and policy makers).	13
Limitations	25	Discuss limitations at study and outcome level (e.g., risk of bias), and at review-level (e.g., incomplete retrieval of identified research, reporting bias).	15
Conclusions	26	Provide a general interpretation of the results in the context of other evidence, and implications for future research.	17
**FUNDING**			
Funding	27	Describe sources of funding for the systematic review and other support (e.g., supply of data); role of funders for the systematic review.	-

For peer review only

Supplement 2: MEDLINE search strategy

1. exp Mental Disorders/ or mental disease*.mp. or exp Depression/ or mental disorder*.mp. or mental illness*.mp. or mental health illness*.mp.
2. mental disorder*.mp. [mp=title, abstract, original title, name of substance word, subject heading word, keyword heading word, protocol supplementary concept word, rare disease supplementary concept word, unique identifier]
3. Exp Mental Health/ or mental health problem*.mp. or exp Stress, Psychological/
4. stress.mp.
5. exp Depression/
6. depression.mp. [mp=title, abstract, original title, name of substance word, subject heading word, keyword heading word, protocol supplementary concept word, rare disease supplementary concept word, unique identifier]
7. exp Anxiety/ or anxiety.mp. or exp Anxiety Disorders/ or anxiety disorder*.mp.
8. (nervous breakdown$ or depression or depressive$ or affective disorder$ or mood disorder$ or anxiety disorder$ or phobia or phobic or panic disorder$ or PTSD or stress disorder$ or OCD or compulsive disorder$).mp. [mp=title, abstract, original title, name of substance word, subject heading word, keyword heading word, protocol supplementary concept word, rare disease supplementary concept word, unique identifier]
9. (anxi* or depress* or melancholi* or neuros* or neurotic or psychoneuro* or stress* or distress* or emotion*).mp.
10. ((mental$ or psychiatric or psychologic$) adj3 (illness$ or condition$ or disabil$ or disorder$ or disease$ or impair$ or problem)).mp. [mp=title, abstract, original title, name of substance word, subject heading word, keyword heading word, protocol supplementary concept word, rare disease supplementary concept word, unique identifier]
11. (bipolar or mania or dissociative disorder$ or neurosis or personality disorder$ or psychosis or paranoi$ or schizophren$ or schizoaffective disorder$ or psychotic).mp. [mp=title, abstract, original title, name of substance word, subject heading word, keyword heading word, protocol supplementary concept word, rare disease supplementary concept word, unique identifier]
12. 1 or 2 or 3 or 4 or 5 or 6 or 7 or 8 or 9 or 10 or 11
13. (Primary adj2 (care or health or healthcare)).mp. [mp=title, abstract, original title, name of substance word, subject heading word, keyword heading word, protocol supplementary concept word, rare disease supplementary concept word, unique identifier]
14. primary health care/ or *continuity of patient care*/ or patient-centred care or progressive patient care/
15. (Primary pratic* or Family pratic* or General practice*).mp. [mp=title, abstract, original title, name of substance word, subject heading word, keyword heading word, protocol supplementary concept word, rare disease supplementary concept word, unique identifier]
16. Community Health Services/
17. community Health Service*.mp. [mp=title, abstract, original title, name of substance word, subject heading word, keyword heading word, protocol supplementary concept word, rare disease supplementary concept word, unique identifier]
18. exp Community Mental Health Services/ or community care.mp. or Community Mental Health Service.mp. [mp=title, abstract, original title, name of substance word, subject heading word, keyword heading word, protocol supplementary concept word, rare disease supplementary concept word, unique identifier]
19. exp Family Practice/ or exp General Practice/
20. 13 or 14 or 15 or 16 or 17 or 18 or 19
21. (mental health adj2 (nurs* or therap* or counsellor* or worker* or practitioner*)).mp. [mp=title, abstract, original title, name of substance word, subject heading word, keyword heading word, protocol supplementary concept word, rare disease supplementary concept word, unique identifier]
22. psychotherapist.mp.
23. therapist*.mp.
24. counsellor*.mp.
25. 21 or 22 or 23 or 24

For peer review only

26. co-locat*.mp. [mp=title, abstract, original title, name of substance word, subject heading word, keyword heading word, protocol supplementary concept word, rare disease supplementary concept word, unique identifier]
27. colocat*.mp. [mp=title, abstract, original title, name of substance word, subject heading word, keyword heading word, protocol supplementary concept word, rare disease supplementary concept word, unique identifier]
28. integrat*.mp. [mp=title, abstract, original title, name of substance word, subject heading word, keyword heading word, protocol supplementary concept word, rare disease supplementary concept word, unique identifier]
29. ((integrat or within or collaborat* or colocat* or co-locat* or inside) adj primary health care).mp. [mp=title, abstract, original title, name of substance word, subject heading word, keyword heading word, protocol supplementary concept word, rare disease supplementary concept word, unique identifier]
30. ((integrat or within or collaborat* or colocat* or co-locat* or inside) adj primary care).mp. [mp=title, abstract, original title, name of substance word, subject heading word, keyword heading word, protocol supplementary concept word, rare disease supplementary concept word, unique identifier]
31. ((integrat or within or collaborat* or colocat* or co-locat* or inside) adj primary healthcare).mp. [mp=title, abstract, original title, name of substance word, subject heading word, keyword heading word, protocol supplementary concept word, rare disease supplementary concept word, unique identifier]
32. exp "Delivery of Health care, Integrated*/ or integrated care.mp. or integrated healthcare.mp. or integrated health care.mp.
33. collaborat*.mp. [mp=title, abstract, original title, name of substance word, subject heading word, keyword heading word, protocol supplementary concept word, rare disease supplementary concept word, unique identifier]
34. 26 or 27 or 28 or 29 or 30 or 31 or 32 or 33
35. 12 and 20 and 25 and 34

For peer review only

Supplement 3: Bias levels in non-randomised studies. Obtained via utilization of the Robins-I tool(15).

	Confounding bias	Bias in selection of participants	Bias in classification of interventions	Bias due to deviations from intended interventions	Bias due to missing data	Bias in measurement of outcomes	Bias in selection of the reported result
Kates et al., (27)	?	?	+	?	+	+	+
Cigrang et al., (26)	−	+	+	+	?	?	+
Abidi et al., (29)	?	+	+	+	?	+	+
Evans et al., (30)	?	?	?	+	?	+	+
Milne and Souter (40)	+	?	+	+	−	+	+
Bridges et al., (38)	+	+	+	+	−	−	+
Magnée et al., (34)	+	+	+	?	?	?	+
Pryde and Jachuck (32)	?	?	+	+	?	?	+
Spurgeon et al., (31)	+	−	?	+	−	+	+
Magnée et al., (33)	?	+	+	+	−	−	+

For peer review only

Supplement 4: Bias levels in randomised controlled trials. Obtained via utilization of the Cochrane bias tool(16).

	Random sequence generation	Allocation concealment	Blinding of participants and personnel	Blinding of outcome assessment	Incomplete outcome data	Selective reporting	Anything else, ideally prespecified
Boot et al., (28)	+	−	−	−	?	?	?
McMahon et al., (35)	?	?	−	+	−	+	?
Lester et al., (36)	+	+	−	?	+	+	?
Marks (37)	?	?	−	?	−	+	?
Friedli, King and Lloyd (39)	?	?	−	−	?	?	?

For peer review only

Supplement 5: Bias assessment for randomised controlled trials, summary of results.

Cochrane Risk of Bias Tool for Randomized Controlled Trials.

Author	Selection bias		Performance bias	Detection bias	Attrition bias	Reporting bias	Other bias	Total
	Random sequence generation	Allocation concealment	Blinding of participants and personnel	Blinding of outcome assessment	Incomplete outcome data	Selective reporting	Anything else, ideally prespecified	
Boot et al., (28)	Low	High	High	High	Unclear	Unclear	Unclear	Poor quality
McMahon et al., (35)	Unclear	Unclear	High	Low	High	Low	Unclear	Fair quality
Lester et al., (36)	Low	Low	High	Unclear	Low	Low	Unclear	Good quality
Marks (37)	Unclear	Unclear	High	Unclear	High	Low	Unclear	Poor quality
Friedli, King and Lloyd (39)	Unclear	Unclear	High	High	Unclear	Unclear	Unclear	Poor quality

For peer review only

Supplement 6: Bias assessment in non-randomised studies of interventions, summary of results. Risk Of Bias In Non-randomized Studies of Interventions (ROBINS-I) tool.

Author	Pre-intervention		At intervention	Post intervention				Overall bias
	Confounding bias	Bias in selection of participants	Bias in classification of interventions	Bias due to deviations from intended interventions	Bias due to missing data	Bias in measurement of outcomes	Bias in selection of the reported result	
Kates et al., (27)	Unclear	Unclear	Low	Unclear	Low	Low	Low	Good Quality
Cigrang et al., (26)	High	Low	Low	Low	Unclear	Unclear	Low	Fair Quality
Abidi et al., (29)	Unclear	Low	Low	Low	Unclear	Low	Low	Good Quality
Evans et al., (30)	Unclear	Unclear	Uncertain	Low	Unclear	Low	Low	Unclear Quality
Milne and Souter (40)	Low	Unclear	Low	Low	High	Low	Low	Fair quality
Bridges et al., (38)	Low	Low	Low	Low	High	High	Low	Good quality
Magnée et al., (34)	Low	Low	Low	Unclear	Unclear	Unclear	Low	Good quality
Pryde and Jachuck (32)	Unclear	Unclear	Low	Low	Unclear	Unclear	Low	Unclear quality
Spurgeon et al., (31)	Low	High	Unclear	Low	High	Low	Low	Fair Quality
Magnée et al., (33)	Unclear	Low	Low	Low	High	High	Low	Fair Quality

For peer review only

Supplement 7: Summary of findings in individual studies

Author	Findings
Kates et al., (27)	Clinical: An average change in score of 17.6 (CESD) and 5.7 (GHQ) was recorded. Furthermore, after comparing the numbers of patients classified as a case (due to exceeding cut off score) before and after treatment, there was 73% (CESD) and 82% (GHQ) less cases after treatment. All changes statistically significant (P<0.005).<br>Service utilization: 65% reduction in referrals to psychiatry outpatient since program started. Nonsignificant reduction of 10% in inpatient admissions.<br>Satisfaction: Overall consumer satisfaction rating of 92% (CSQ). Counsellors and practitioners satisfied.
Cigrang˙ et al., (26)	Clinical: OQ-45 scores decreased significantly for patients with more than one appointment. There was also a significant reduction in OQ-45 scores between patients that had either had 1, 2 or 4 or more appointments. This was also maintained during follow up (P range 0.032-0.001)<br>Service utilization: Significant increase in medical appointments P<0.01.
Boot et al., (28)	Clinical: Post intervention GHQ scores were significantly reduced for both the counselling intervention group and a control GP advice group. However, GHQ scores were significantly lower in the intervention group than in the control group and the average decrease in scores was twice as high in intervention group. There was a significantly larger proportion of the control group scoring above the GHQ threshold level post study, 46% compared to 25%.<br>Psychotropic drug usage:<br>Significantly fewer prescriptions during the 6- week period in the counselled group compared to GP care. Similar finding for only antidepressants but not anxiolytic drugs.<br>Referrals and consultation rates: Significantly higher referrals in GP group. No significant differences in follow-up consultations rates.<br>Satisfaction: significantly more counselled patients reported coping better with their problem, feeling happier, satisfied with treatment and had enough time to talk.
Abidi et al., (29)	Diagnoses: significant decrease of chronic alcohol abuse and acute alcohol abuse diagnoses for all groups (control, only PN-MH, only PCP, both PCP and PNMH). both PCP and PNMH higher mean of chronic and acute alcohol abuse diagnoses than control practices. No significant differences were found between groups.
Evans et al., (30)	Referrals: mean referral rate of BME patients for GP practices without a link worker 0.35 per week per 10000 patients and was unchanged throughout study. Referral rates for practices with a link worker increased from 0.65 to 1.37 referrals per week per 10000 patients.
Spurgeon et al., (31)	Clinical outcomes: All patients showed reductions in subjective distress (CORE-OM), excluding the diabetes group, and was maintained post-intervention. The HADS anxiety and depression scales show there was significant reductions in anxiety in all patient groups, patients with hypertension and frequent attenders had reductions in depression also. The total patient sample achieved significant reductions in the majority of SF36 subscales, excluding diabetic patients<br>Uptake of health services: Counselling intervention significantly reduced uptake of health services
Pryde and Jachuck (32)	Clinical outcomes: After an average of three consultations, out of 84 patients, 44% were considered to be much improved, 31% improved and 25% unchanged in terms of symptomatic severity and prevalence.
Magnée et al., (34)	Service utilization: GPs in practices with a mental health nurse have similar consultation rates than GPs without one. GPS with a mental health nurse have more patients exhibiting psychological or social disorders (OR=1.05). Nurses do not seem to replace GP care, but probably deliver additional consultations to patient

For peer review only

McMahon et al., (35)	Clinical outcomes: Little difference between the two treatments BDI. Although, significant gradual symptomatic improvement was seen over time for this measure. Some improvement over time was seen for other measures (HDRS17, MADRS and SASS) but was not significant and could be as a result of intervention group patients being significantly more likely to have medication adjustment (P<0.01).<br>Satisfaction: Patient satisfaction high for both treatments.
Lester et al., (36)	Clinical outcomes: Although there was an average improvement in patient symptoms in the intervention group there was not enough evidence to suggest this was significant (CORE-OM), a difference in means of -0.35 (CI 0.52 to -1.22).<br>Satisfaction: Intervention practices had higher satisfaction levels<br>Voluntary sector usage: No difference in usage between groups
Marks (37)	Clinical outcomes: Intervention patients obtained significantly improved results than those on the wait list and under general practitioner care. There was also a high level of agreement in diagnosis and patient care management between nurse and a psychiatrist (used to assess if treatments were correct).
Bridges et al., (38)	Clinical Outcomes: Using ACORN, it was found that both Latinos and Whites had significant clinical improvement and there were no significant symptom improvement differences between them.<br>Service utilization: Non-Latinos have significantly increased post intervention telephone service utilization rates than Latinos. No difference in in-person visits.<br>Attendance of follow-up appointments: no significant differences in follow-up rates or attendance of appointments.<br>Satisfaction: Latino patients significantly more satisfied with BHC and first visits.
Friedli, King and Lloyd (39)	Clinical: There were no significant differences between the groups on any of the mental health outcomes at three or nine months. (BDI, BSI, CRCIS)<br>Costs: Direct and indirect counsellor costs are<br>£162 more per patient compared to GP care (3-months). However, the counsellor costs were £87 less per patient for the preceding 6 months. Overall counsellor group was more expensive.
Milne and Souter (40)	Clinical outcomes: There was a significant increase in active cognitive coping skills and a significant decrease in strain levels after 3 months (P<0.05).<br>Costs: Overall increase in drug costs over the period. The costs of psychotropic and other CNS drugs reduced in the improved coper group, whereas increased significantly in the other group. Reductions<br>in costs for the improved copers were due less drug usage, less hospital referrals and less GP visits
Magnée et al., (33)	Prescription effects: Although, there was an initial drop in prescription rates, mental health nurses in general practice did not decrease the overall prescription rates of antidepressants.

For peer review only

Synthesis Without Meta-analysis (SWiM) reporting items

The citation for the Synthesis Without Meta-analysis explanation and elaboration article is: Campbell M, McKenzie JE, Sowden A, Katikireddi SV, Brennan SE, Ellis S, Hartmann-Boyce J, Ryan R, Shepperd S, Thomas J, Welch V, Thomson H. Synthesis without meta-analysis (SWiM) in systematic reviews: reporting guideline BMJ 2020;368:l6890 http://dx.doi.org/10.1136/bmj.l6890

**SWiM is intended to complement and be used as an extension to PRISMA**			
**SWiM reporting item**	**Item description**	**Page in manuscript where item is reported**	**Other***
Methods			
**1** Grouping studies for synthesis	1a) Provide a description of, and rationale for, the groups used in the synthesis (e.g., groupings of populations, interventions, outcomes, study design)	6	
	1b) Detail and provide rationale for any changes made subsequent to the protocol in the groups used in the synthesis		No changes were made to the groups used in the synthesis after protocol establishment
**2** Describe the standardised metric and transformation methods used	Describe the standardised metric for each outcome. Explain why the metric(s) was chosen, and describe any methods used to transform the intervention effects, as reported in the study, to the standardised metric, citing any methodological guidance consulted		No standardised metrics were utilised.
**3** Describe the synthesis methods	Describe and justify the methods used to synthesise the effects for each outcome when it was not possible to undertake a meta-analysis of effect estimates	7	
**4** Criteria used to prioritise results for	Where applicable, provide the criteria used, with supporting justification, to select the particular studies, or a particular study, for the main synthesis or to draw conclusions from the		N/A- no particular measures

For peer review only - http://bmjopen.bmj.com/site/about/guidelines.xhtml

Synthesis Without Meta-analysis (SWiM) reporting items

summary and synthesis	synthesis (e.g., based on study design, risk of bias assessments, directness in relation to the review question)		were prioritised
**SWiM reporting item**	**Item description**	**Page in manuscript where item is reported**	**Other***
**5** Investigation of heterogeneity in reported effects	State the method(s) used to examine heterogeneity in reported effects when it was not possible to undertake a meta-analysis of effect estimates and its extensions to investigate heterogeneity	7	
**6** Certainty of evidence	Describe the methods used to assess certainty of the synthesis findings	7	
**7** Data presentation methods	Describe the graphical and tabular methods used to present the effects (e.g., tables, forest plots, harvest plots).<br>Specify key study characteristics (e.g., study design, risk of bias) used to order the studies, in the text and any tables or graphs, clearly referencing the studies included	7	
Results			
**8** Reporting results	For each comparison and outcome, provide a description of the synthesised findings, and the certainty of the findings. Describe the result in language that is consistent with the question the synthesis addresses, and indicate which studies contribute to the synthesis	8	
Discussion			

For peer review only

For peer review only - http://bmjopen.bmj.com/site/about/guidelines.xhtml

Synthesis Without Meta-analysis (SWiM) reporting items

**9** Limitations of the synthesis	Report the limitations of the synthesis methods used and/or the groupings used in the synthesis, and how these affect the conclusions that can be drawn in relation to the original review question	15	

PRISMA=Preferred Reporting Items for Systematic Reviews and Meta-Analyses.
*If the information is not provided in the systematic review, give details of where this information is available (e.g., protocol, other published papers (provide citation details), or website (provide the URL)).

For peer review only
